# Ecdysteroids are present in the blood of wild passerine birds

**DOI:** 10.1038/s41598-019-53090-9

**Published:** 2019-11-18

**Authors:** Sándor Hornok, Attila Csorba, Dávid Kováts, Tibor Csörgő, Attila Hunyadi

**Affiliations:** 1 Department of Parasitology and Zoology, University of Veterinary Medicine, 1078 Budapest, Hungary; 2 Institute of Pharmacognosy, Interdisciplinary Excellence Centre, University of Szeged, 6720 Szeged, Hungary; 3 Ócsa Bird Ringing Station, 2364 Ócsa, Hungary; 4 Hungarian Biodiversity Research Society, 1165 Budapest, Hungary; 5 Department of Anatomy, Cell- and Developmental Biology, Eötvös Loránt University, 1117 Budapest, Hungary

**Keywords:** Hormones, Metabolism

## Abstract

Ecdysteroids (arthropod molting hormones) play an important role in the development and sexual maturation of arthropods, and they have been shown to have anabolic and “energizing” effect in higher vertebrates. The aim of this study was to assess ecdysteroid diversity, levels according to bird species and months, as well as to observe the molting status of hard ticks (Acari: Ixodidae) infesting the birds. Therefore, blood samples and ticks were collected from 245 birds (244 songbirds and a quail). Mass spectrometric analyses showed that 15 ecdysteroids were regularly present in the blood samples. Molting hormones biologically most active in insects (including 20-hydroxyecdysone [20E], 2deoxy-20E, ajugasterone C and dacryhainansterone) reached different levels of concentration according to bird species and season. Similarly to ecdysteroids, the seasonal presence of affected, apolytic ticks peaked in July and August. In conclusion, this study demonstrates the presence of a broad range and high concentrations of ecdysteroids in the blood stream of wild-living passerine birds. These biologically active, anabolic compounds might possibly contribute to the known high metabolic rate of songbirds.

## Introduction

Steroids are biologically active organic molecules widely present in multicellular eukaryotes. These compounds play an important role in the regulation of reproduction, development and responses to environmental stimuli of plants, fungi and animals. Concerning the latter, while vertebrates synthesize different classes of steroids (e.g. sexual hormones, corticosteroids), arthropods are known to produce a single type of these molecules, the so-called ecdysteroids or molting hormones^1^.

Interestingly, the structure of steroid hormone receptors is highly conserved among metazoans^2^. Although ecdysteroids do not necessarily bind to homologous receptors in different phyla of the Animal Kingdom, they may have a variety of effects even in distantly related taxa. These compounds play an important role in molting and development of pre-adult stages, as well as in the sexual maturation of adult arthropods^3^, but they were also experimentally shown to have anabolic effect in mammals^4^ and galliform birds^5^. Moreover, ecdysteroids are present in plants as phytoecdysteroids, to deter plant-eating insects and phytoparasitic nematodes^6^.

The archetypal ecdysteroid both in arthropods and in plants is 20-hydroxyecdysone (20E), but a broad range of structural analogues are also known to exist^4^. Ecdysteroids naturally occur in mammalian tissues as a consequence of dietary intake of ecdysteroid-containing plants or insects^4^. Recently, a similar phenomenon has been reported among birds. Based on a limited number of samples, it was demonstrated that certain naturally acquired ecdysteroids may reach high levels in the blood of insectivorous passerine birds^7^. In this context, passerine birds appeared to be particularly suitable subjects to study, because caterpillars predominate in their diet^8,9^, and caterpillars are known for their relatively high ecdysteroid concentrations^10^. The results also indicated that these exogenous molting hormones may affect bird ticks by inducing on-host apolysis (which does not take place physiologically among three-host ticks) and thereby possibly reducing the period of tick blood feeding and thus the chances of tick-borne pathogen transmission^7^.

The aim of this study was to broaden the scope of these preliminary observations and to examine their relevance in a broader context: i.e., to evaluate ecdysteroid diversity, peak levels and potential effects on bird-infesting ticks (a) in a larger set of bird samples, and (b) with a more extended and more sensitive mass spectrometric analysis.

## Results

### Blood ecdysteroid diversity according to bird taxa

**Figure 1:**
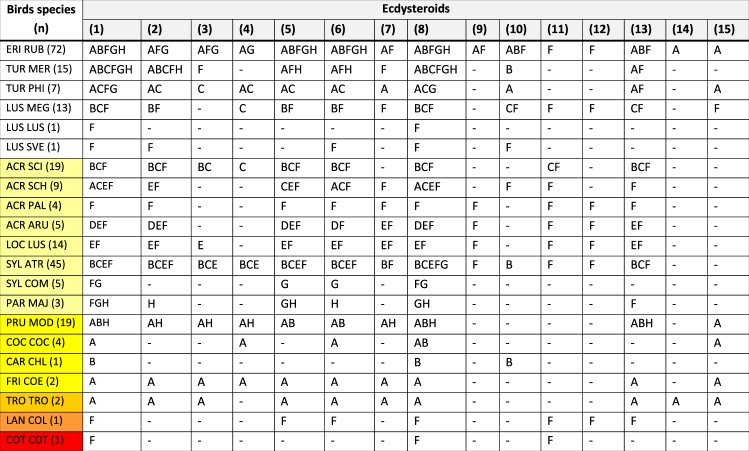
Presence of ecdysteroids in the blood according to month and bird species. In the column headings ecdysteroids are numbered in parentheses as in the text. Data of 243 birds are included, because the species of one bird was not recorded. Color code of left column: white (Muscicapaoidea: Muscicapidae, Turdidae), light yellow (Sylvioidea: Acrocephalidae, Locustellidae, Sylviidae, Paridae), dark yellow (Passeroidea: Prunellidae, Fringillidae), light orange (Troglodytidae), dark orange (Laniidae), red (Galliformes). Abbreviations of birds species: ACR ARU - *Acrocephalus arundinaceus*; ACR PAL - *Acrocephalus palustris*; ACR SCH - *Acrocephalus schoenobaenus*; ACR SCI - *Acrocephalus scirpaceus*; CAR CHL - *Carduelis chloris*; COC COC - *Coccothraustes coccothraustes*; COT COT - *Coturnix coturnix*; ERI RUB - *Erithacus rubecula*; FRI COE - *Fringilla coelebs*; LAN COL - *Lanius collurio*; LOC LUS - *Locustella luscinioides*; LUS LUS - *Luscinia luscinia*; LUS MEG - *Luscinia megarhynchos*; LUS SVE - *Luscinia svecica*; PAR MAJ - *Parus major*; PRU MOD - *Prunella modularis*; SYL ATR - *Sylvia atricapilla*; SYL COM - *Sylvia communis*; TRO TRO - *Troglodytes troglodytes*; TUR MER - *Turdus merula*; TUR PHI - *Turdus philomelos*. Abbreviations of months: A-March, B-April, C-May, D-June, E-July, F-August, G-September, H-October.

**Figure 2:**
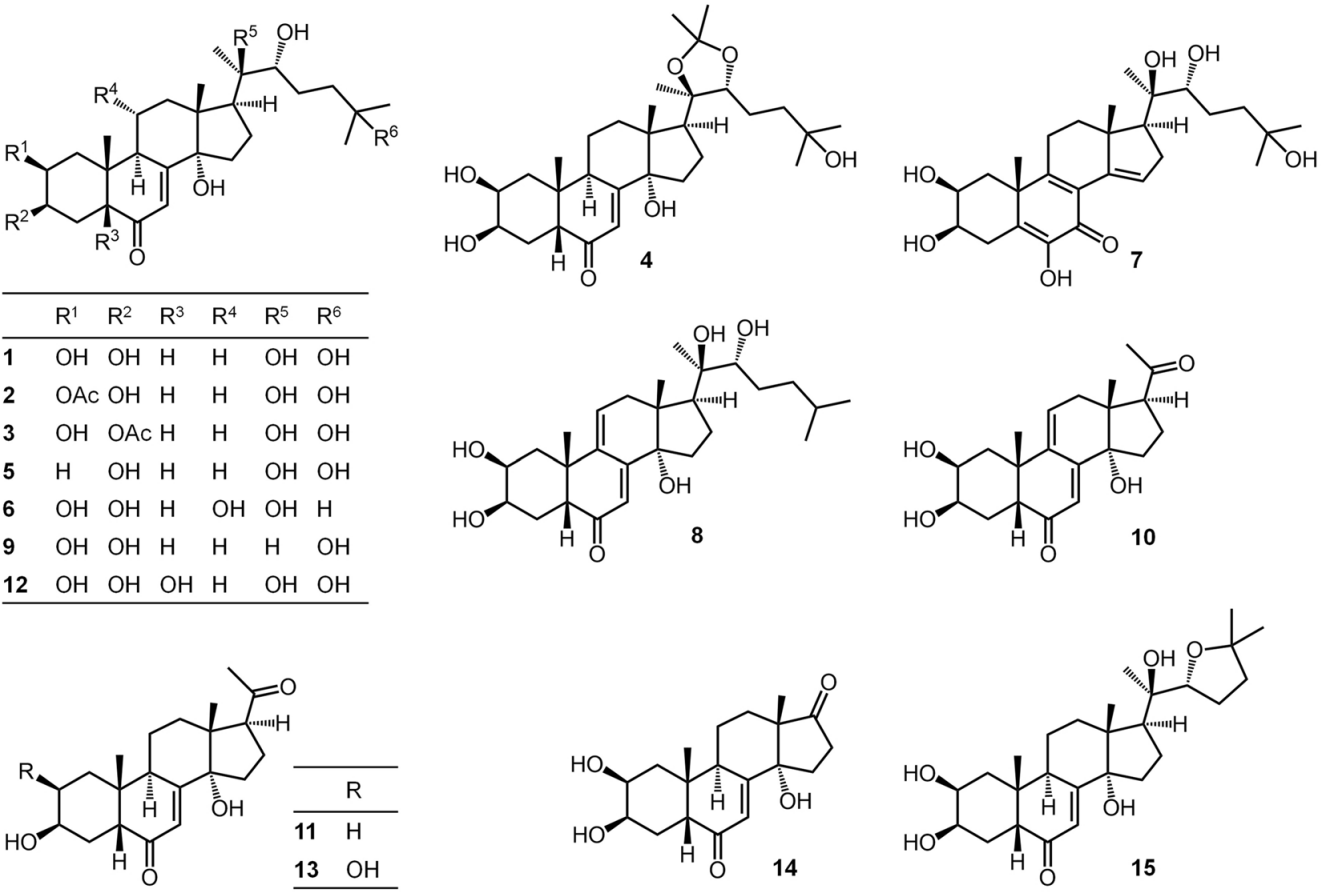
Chemical structures of the ecdysteroids present in the blood samples of passerine birds. 20-hydroxyecdysone (20E; **1**), 20E 2-acetate (**2**), 20E 3-acetate (**3**), 20E 20,22-acetonide (**4**), 2-deoxy-20E (**5**), ajugasterone C (**6**), calonysterone (**7**), dacryhainansterone (**8**), ecdysone (**9**), 9,11-didehydropoststerone (**10**), 2-deoxypoststerone (**11**), polypodine B (**12**), poststerone (**13**), rubrosterone (**14**), shidasterone (**15**).

Among the twenty-five compounds tested for, fifteen ecdysteroids (**1**–**15**) were shown to be present in 244 individuals of 20 songbird (Aves: Passeriformes) species (Fig. 1). In addition, two ecdysteroids, dacryhainansterone (**8**) and 2-deoxypoststerone (**11**) were detected in a quail (Aves: Galliformes). Among the molting hormones biologically most active in insects, i.e. “cardinal ecdysteroids” such as 20E (**1**), 2deoxy-20E (**5**), ajugasterone C (**6**) and dacryhainansterone (**8**), all four have been detected over the whole evaluation period (March to October) in the majority of bird species (Fig. 1). On the other hand, some bird species differed from others in the seasonality of cardinal ecdysteroids: 2-deoxy-20E (**5**) and ajugasterone C (**6**) were absent in samples of *Turdus merula* during most of the spring and summer months, while these compounds were present in samples of *T. philomelos* and *Prunella modularis* only at the beginning of the sampling period (Fig. 1). The presence of certain ecdysteroids appeared to be superfamily-dependent, e.g. rubrosterone (**14**) and shidasterone (**15**) were demonstrated among Muscicapaoidea and Passeroidea, but not in Sylvioidea. At the same time, ecdysone (**9**), 2-deoxypoststerone (**11**) and polypodine B (**12**) were present in Muscicapaoidea and Sylvioidea, unlike in Passeroidea (Fig. 1). As to the results obtained for compounds **4** and **18**, it is worth mentioning that the ideal HPLC solvent system was an acid-containing one, which therefore may have catalyzed the elimination of 2,3-acetonide groups. Accordingly, while 20E 2,3;20,22-diacetonide (**18**) was not detected in any of the samples, this may be the result of its decomposition to 20E 20,22-acetonide (**4**) during the analysis. The chemical structures of compounds **1**–**15** are presented in Fig. 2.

### Cardinal blood ecdysteroid concentrations according to months

**Figure 3:**
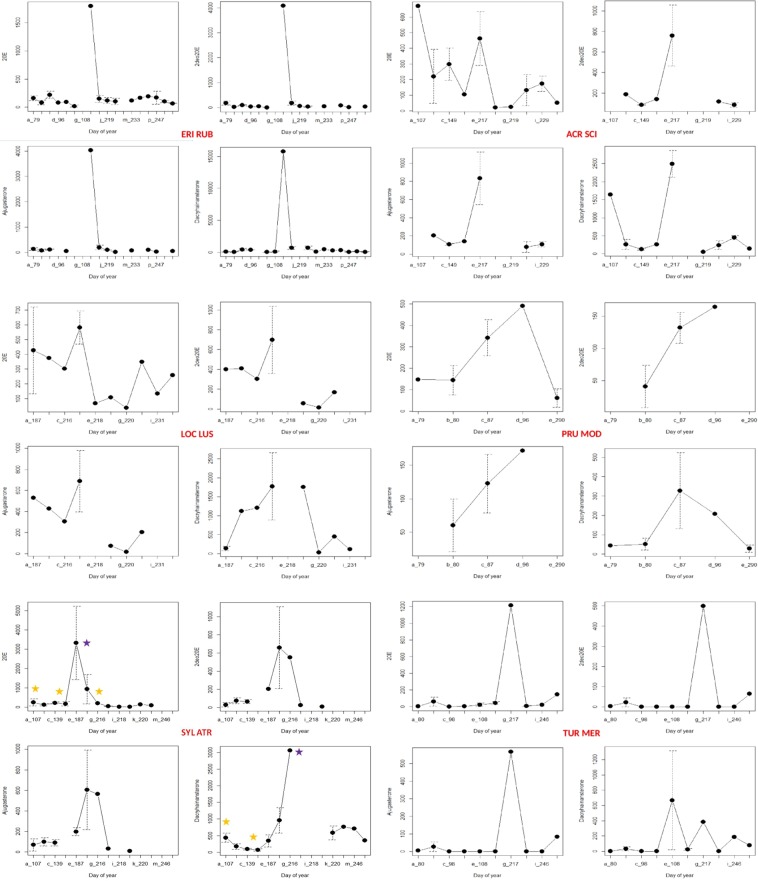
Mean hormone levels (vertical axis: ng/mL) calculated for the cardinal ecdysteroids in the case of six bird species providing the majority of samples. Days of the year as sampling times (horizontal axis) are detailed in Supplementary File 4, according to bird species and months. Purple star indicates significantly higher concentration compared to values marked with yellow star. For abbreviations of bird species see Fig. 1.

Seasonality of cardinal ecdysteroids was analyzed in the six bird species providing the majority (n = 185) of samples. In *Erithacus rubecula*, the highest concentrations of all four cardinal ecdysteroids was measured in the blood of one individual sampled in August (Fig. 3). Similarly, all four cardinal ecdysteroids peaked in August in *Acrocephalus scirpaceus* (although 20E had another peak in April), *Locustella luscinioides* and *Turdus merula* (although dacryhainansterone had another peak in April) (Fig. 3). However, in *Prunella modularis*, the four cardinal ecdysteroids peaked in the spring, March to April (Fig. 3). Hormone levels peaked in July and August for *Sylvia atricapilla*, with significantly higher levels compared to subsequent and/or previous months in the case of 20E and dacryhainansterone (20E: April, May and August *vs* July, F = 8.158, df = 3, P = 0.0003; April *vs* July, t = 4.793, SE = 661.16, P < 0.001; May *vs* July, t = −3.892, SE = 802.36, P = 0.002; August *vs* July, t = 4.400, SE = 690.56, P = 0.0004; dacryhainansterone: April and May *vs* August, F = 4.788, df = 4, P = 0.003; April *vs* August, t = 4.055, SE = 219.0, P = 0.002; May *vs* August, t = −3.572, SE = 292.0, P = 0.008) (Fig. 3).

### Cardinal blood ecdysteroid concentrations according to tick apolysis

**Figure 4:**
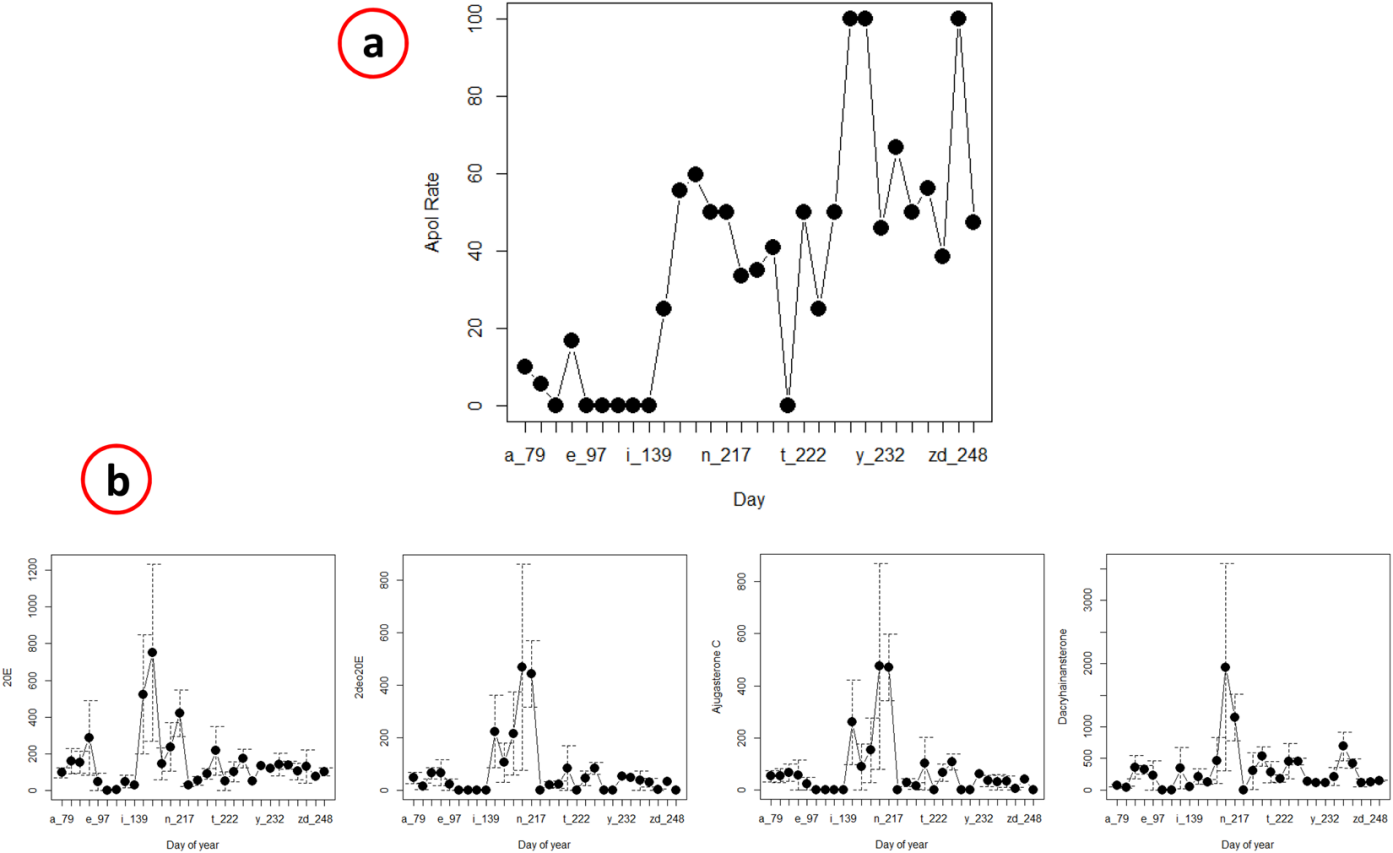
(**a**) Percentage of ticks with apolysis (vertical axis) according to days of the year when sampling was performed (horizontal axis). The percentage was calculated by dividing the number of tick(s) showing apolysis with the number of all ticks collected from the same bird individual, and the result multiplied by one hundred. These data are detailed in Supplementary Table S1, according to bird species and months. (**b**) The corresponding concentrations (vertical axis: ng/mL) of the cardinal ecdysteroids calculated for the same days.

Ticks were found on 115 birds, belonging to 17 species (Table S1). Compared to spring time (when only four birds had apolytic ticks vs 33 birds had no apolytic ticks), significantly (P < 0.0001) higher number of birds had apolytic than non-apolytic ticks both in the summer (42 vs 20) and in the autumn (12 vs four). Taken together, the rate of apolysis had three peaks among these ticks: in July, August and September (Fig. 4a). Concentrations of cardinal ecdysteroids in these birds had a similar tendency, i.e. peaked in August (and there was another peak of 20E in July: Fig. 4b). In the case of individual birds, no significant correlation was found between cardinal ecdysteroid concentrations in their blood and the ratio of their apolytic ticks. However, when the percentage of birds with apolytic ticks was considered according to days of the year, there was a nearly significant correlation with ajugasterone C (**6**) (R = 0.180, P = 0.055) and dacryhainansterone (**8**) concentration (R = 0.183, P = 0.05).

## Discussion

In this study, the frequent and natural occurrence of low to high concentrations of a broad range of ecdysteroids was demonstrated in passerine birds, and one galliform bird, for the first time. The peak levels of the four cardinal hormones were nearly unanimously observed around August. Seasonal differences of peak concentrations of ecdysteroids according to bird species might be associated with their feeding preferences or differences in habitat. For instance, in the nesting period the preferred food items of passerine birds are caterpillars^8,9^, which have peak abundance around early summer in Hungary (depending on their species^11^), i.e. preceding peak ecdysteroid levels relevant to most bird species. In another study, peak abundance of caterpillars was observed between the 117th and 188th days of the year, depending on year and biotope type^12^. This broad range includes the first peak of blood ecdysteroids and apolytic ticks (day 187: Fig. 4, Table S1), meaning correspondence with the data presented here. On the other hand, dipterans (e.g. Muscidae) have overwintering larvae already active from February^13^. The latter may partly explain early peaking of ecdysteroids in *P. modularis*, which in the spring time predominantly feeds on dipterans^14^. In addition, several song bird species investigated here are known to feed on plants (fruits, grains), particularly towards the autumn/winter, when insects become less available^15^. In this way, plant-derived ecdysteroids may have also contributed to the high compound levels observed here, as also evident from the facts that neither ajugasterone C nor dacryhainansterone are known to naturally occur in arthropods, and the latter is currently only recognized as a phytoecdysteroid^6^. On the other hand, one could hypothesize that these phytoecdysteroids might also reach insectivorous birds indirectly, i.e. through the plant-caterpillar-bird food chain.

The pharmacokinetics of 20E (**1**) depend on the mode of administration. For example, the half-time of elimination of this substance in lambs were 0.2, 0.4, and 2 h when administered *via* oral, intravenous, and intramuscular routes, respectively^16^. Such data have not hitherto been available for passerine birds, but supposing the lowest half-time for oral uptake similarly to mammals, the high concentrations detected in this study suggest (a) continuous access to ecdysteroids from insects, and/or (b) their slower metabolism by birds. Under experimental conditions the blood levels of 20E were proportional to the amounts given orally in galliform birds^5^, suggesting a slow metabolism or elimination of this compound. The high concentrations of the four cardinal ecdysteroids observed here also support this.

20E has low toxicity in mammals (the LD50 exceeds 9 g/kg after oral administration^17^). At the same time, ecdysteroids have a variety of beneficial physiological effects in mammals, including anabolic, hepatoprotective, immune modulatory, as well as hypoglycemic action^4^. Notably, 20E stimulates certain major metabolic pathways such as protein synthesis, and lipid, carbohydrate metabolism^18^. One of the most important properties of ecdysteroids in mammals is their anabolic effect (similar to anabolic steroids, but without the androgenic effect) and that they enhance physical performance^19,20^. In particular, the anabolic effect of ecdysteroids as natural substances on muscles can exceed that of the well-known anabolic substances, and ecdysone is known to enhance physical performance (acting as a “natural dope“^21^).

The latter is especially relevant to birds, taking into account their high metabolic rate and energy need for flying. Similarly to mammals but to a lesser extent, in birds the anabolic activity of orally administered ecdysteroids is documented: these substances improve growth rate and disease resistance in galliform birds^5,22,23^. Therefore, the potential of ecdysteroids to increase energy levels and to enhance physical performance can also be postulated in avian hosts.

Among avian orders, members of Passeriformes are known for their highest energy levels and intense metabolic rates^24,25^, in association with their activities, habitats (even in high altitudes), cold hardiness (winter survival) or, alternatively, seasonal migrations^24,25^. Migration and winter acclimatization are intimately linked with the metabolic properties of the highly aerobic skeletal muscle contained within the flight apparatus of passerines^24^. In this way the abundance of ecdysteroids in their blood, as shown here, might be highly beneficial, taking into account the anabolic action of these hormones (i.e., stimulating muscle build-up and physical performance).

Concerning ectoparasites, ecdysteroids have a similar role in ticks as in other arthropod groups, i.e. they trigger and control molting^26^. In the case of three-host ticks (including those collected during this study), sharp rise of endogenous ecdysteroid levels can be observed only after detachment from the host, with contemporaneous induction of apolysis (cuticle detachment, the initial phase of molting)^26^. Therefore, under such circumstances, apolysis takes place exclusively in the environment. On the contrary, when ticks are provided with ecdysteroid-containing blood (i.e. as an exogenous source of molting hormones), it may accelerate their development and may induce apolysis in a dose-dependent way^27^.

However, dose-dependency of this phenomenon could not have been demonstrated here, because there was no consistent correlation between ecdysteroid concentrations in a particular individual bird and the apolytic state of its ticks (especially between days 217–248: Fig. 4). This can be partly attributed to the unknown feeding status of these ticks (i.e., for how long they have been exposed to blood-borne ecdysteroids prior to their collection). Apart from the dose, on-host induced apolysis of a tick may also depend on the type of ingested ecdysteroid and on the tick species itself. For instance, *Ornithodoros moubata* is able to inactivate (via conjugation with fatty acids) ingested ecdysone and 20E, but not 22,25-dideoxyecdysone^28^; whereas in *O. porcinus* feeding on 20E-containing blood accelerated molting^27^. Importantly, to the best of our knowledge, no information is available on the ecdysteroid metabolism of the two tick species (*Ixodes ricinus*, *Haemaphysalis concinna*: abundance data not shown) involved here.

Nevertheless, the present data reflected an overall tendency that apolytic ticks, as well as peak ecdysteroid titers are seasonally associated with summer to early autumn period. This confirms our previous results^7^, but on a much larger sample size. It also has to be taken into account that in our previous study less than half (eight of 18 = 44%) of birds had detectable ecdysteroid titres, and only two of them (11.1%) were shown to contain ajugasterone C^7^. This may be partly explained by the limited sample size and by the lower sensitivity of the detection method used previously.

In conclusion, this is the first study to demonstrate the presence of a broad range of ecdysteroids in the blood stream of wild living passerine birds, with considerable variation of blood concentrations according to bird species and season, sometimes reaching very high levels. These biologically active, anabolic compounds might possibly contribute to the known high metabolic rate of songbirds.

## Methods

### Ethical approval

All international, national, and institutional guidelines established for the care of wild birds were followed. The experimental protocol (blood sampling) was approved by the committee of the Middle -Danube -Valley Inspectorate for Environmental Protection, Nature Conservation and Water Management (under registration number KTF: 27251-1/2014).

### Sample collection and preparation

Sample collection was performed in 2014 (March to October) at Ócsa Bird Ringing Station (47° 17′ 54.3″ N, 19° 13′ 52.1″ E). Birds were captured by standard Ecotone mist-nets (Gdynia, Poland), 12 m in length, 2.5 m in height and with 16 × 16 mm mesh. From 245 individuals, blood samples were collected into EDTA-containing microtubes from the brachial vein using a fine (28G) needle and 0.5 mL syringe (Kendall Monoject: Tyco Healthcare Group Lp., Mansfield, MA, USA). These samples were kept frozen at −20 °C. The whole body of each captured bird was also scrutinized for the presence of ticks. All ticks were removed with fine tweezers, and put into 70% ethanol in separate vials according to their hosts. The species of ticks were identified using standard morphological keys. The state of cuticle detachment (apolysis) among ticks was assessed using a stereo microscope (SMZ-2 T, Nikon Instruments, Japan, illuminated with model 5000–1, Intralux, Urdorf-Zürich, Switzerland).

Sample preparation was performed as we published before^7^. Briefly, one hundred or 250 μL of physiological saline solution was added to the frozen blood samples, and, after carefully homogenizing, each was transferred to Eppendorf tubes with a Hamilton syringe. The volume increment as compared to that of the added volume was considered as the original volume of blood. Following this, the same amount of methanol was added, the solution was homogenized by shaking and left at room temperature for at least half an hour. The precipitate was subsequently centrifuged at 10,000 rpm for 10 min at 8 °C, and the clear supernatant was utilized for LC-MS/MS studies.

### LC-MS/MS analysis

The quantitative analysis of the biological samples was performed on Agilent 1100 HPLC system (Santa Clara, USA) coupled with Thermo Scientific Q Exactive Plus orbitrap mass spectrometer (Waltham, MA USA). The HPLC consisted of binary pump, thermostated autosampler and thermostated column compartment. The column used was Kinetex XB-C18 (particle size 2.6 µm, pore size 100 Å, length/diameter in mm: 100/2.1) from Phenomenex (Gen-Lab Kft., Budapest, Hungary). The gradient elution was starting from 15% eluent B increased to 35% in 20 minutes at 0.5 mL/min flow rate where eluent A and B was MS grade water and acetonitrile, respectively, both containing 0.1% formic acid (VWR International Kft., Debrecen, Hungary). The gradient was followed with 10 minutes wash with 90% eluent B and then the column was equilibrated with 15% eluent B for 10 minutes. The mass spectrometer operated in positive MS mode with HESI source (spray voltage: 3500 V, capillary temperature: 300 °C, sheath gas/auxiliary gas/spare gas: 55/15/5 in arbitrary units, sheath gas temperature: 400 °C). The compounds were identified by their exact mass and unique retention time. LOD and LOQ criteria were defined as the signal to noise ratio of 3 and 10, respectively. A description of the method development process and a detailed summary of the identification parameters can be found in the Supporting Information, S1 Text, and ions chosen for the data acquisition for each compound are listed in Table S2.

### Calibration

Standard ecdysteroids 20-hydroxyecdysone (20E; **1**), 20E 2- and 3-acetate (**2** and **3**, respectively), 20E 20,22-acetonide (**4**), 2-deoxy-20E (**5**), ajugasterone C (**6**), calonysterone (**7**), dacryhainansterone (**8**), ecdysone (**9**), 9,11-didehydropoststerone (**10**), 2-deoxypoststerone (**11**), polypodine B (**12**), poststerone (**13**), rubrosterone (**14**), shidasterone (**15**), 20E 22-acetate (**16**), 5α-20E (**17**), 20E 2,3;20,22-diacetonide (**18**), 5β-hydroxypoststerone (**19**), 11α-hydroxypoststerone (**20**), the 17β-acyl analog of calonysterone (**21**), ajugalactone (**22**), cyasterone (**23**), herkesterone (**24**), and 3-*epi*-20E (**25**) were obtained from our previous phytochemical studies^29–32^. Compounds **1** and **4**–**25** possessed a purity of >95%. Compounds **2** and **3** were present as a mixture, and, since ecdysteroid 2- and 3-acetates are able to interconvert in solution^33,34^, calibration for these two compounds was performed with the same standard by considering the calculated peak area ratio of 0.23642: 0.76358 (SD = 0.010586) for compounds **2** and **3**, respectively. This ratio was found stable during the course of our study. Elution order of these two compounds could be unambiguously established based on our previous related work^31^.

Standard stock solutions of the compounds were prepared in methanol at different concentration levels depending on the availability. The working standard solution was obtained by mixing the stock solutions and further 100-fold dilution in methanol gave the highest calibration level. 7 calibration levels were serial diluted with methanol in a 1:3 pattern. All prepared solutions were stored at 4 °C before use. The biological samples contained low levels of the ecdysteroids, hence the calibration was finally fitted to the lowest 5 levels three replicates at each levels. Concentrations of the stock solution and the calibration levels are presented in the Supporting Information, Table S3, and an example chromatogram of the calibration is shown in Fig. S1. All calibration curves were fitted linearly with an R^2^ > 0.995, and a detailed summary of each individual calibration can be found in the Supporting Information, Table S4. In the case of compound **11**, nine samples contained higher concentrations than that of calibration level L5, and fell out of the linear range. These are marked as “ALOQ” (above limit of quantification) in Table S6.

### Statistical analyses

The presence or absence of apolytic ticks on birds was compared according to seasons with Fisher’s exact test. Based on their experimentally shown biological activity in arthropods, 20E (**1**), 2-deoxy-20E (**5**), ajugasterone C (**6**) and dacryhainansterone (**8**) (referred to here as cardinal ecdysteroids) were selected as the primary targets of statistical comparison, also taking into account their presence in the majority of bird individuals throughout the evaluation period. These four cardinal ecdysteroids have high efficacy in triggering molting, as demonstrated by their –log(ED50[M]) values of 6.18–9.28 *in vitro* in the Drosophila B_II_ bioassay^35^. For statistical comparison of mean of each variable, linear model (Lm) and for post-hoc pairwise comparison Tukey-test were used. The variability was analyzed with Levene’s test. All the calculations were done using the statistical software R 3.1.1.^36^. The level of significance was set to 0.05.

## Supplementary information

Supplementary Information

## Data Availability

All data used in the study are included in the manuscript and in the Supplementary Files.
