# Supplementary material for: Ecdysteroids are present in the blood of wild passerine birds
Source: Sci Rep. 2019 Nov 18;9:17002. doi: 10.1038/s41598-019-53090-9 (PMC6861316; doi:10.1038/s41598-019-53090-9)
Supplement: Supplementary file 1 — Supplementary Information [file 41598_2019_53090_MOESM1_ESM.pdf]

Ecdysteroids are present in the blood of wild passerine birds

Sándor Hornok, Attila Csorba, Dávid Kováts, Tibor Csörgő, Attila Hunyadi

SUPPLEMENTARY INFORMATION

S1 Text

LC-MS/MS method development

The same LC method gradient range was used as in our previous study (Hornok et al, Sci Rep, 2016), but the gradient time was increased in order to better suit the longer column and the need for separating a higher diversity of the target compounds than before. The optimal length of the gradient time was selected based on the criterion of baseline separation for the isobaric compounds.

During the MS method development it had to be considered that the ecdysteroids have a high structure similarity thus their fragmentation provides similar product ions, and that there is a high chance for in source fragmentation (multiple water losses) due to the high hydroxyl group content. Due to these facts there is no unique precursor ion for most of the compounds, and, due to the multiple water losses, the product ion m/z values show overlap between the different compounds. Based on these, the acquisition mode was optimized to positive ESI MS scan mode, and the quantitative information was produced by the summed extracted ion chromatograms of the given precursor and its neutral losses. The quantitative ions for the compounds were determined by infusing the pure reference compound solution (one by one) to 500 µl/min LC flow through a TEE-connector so the in-source fragmentation pattern was equivalent, which was expected during the LC method conditions. For a summary of the ions, see Table S2.

Figure S1. Example chromatogram of the calibration level 5. Retention time shift was allowed to 0.1 min. Identification of the peaks are included in Table S1.

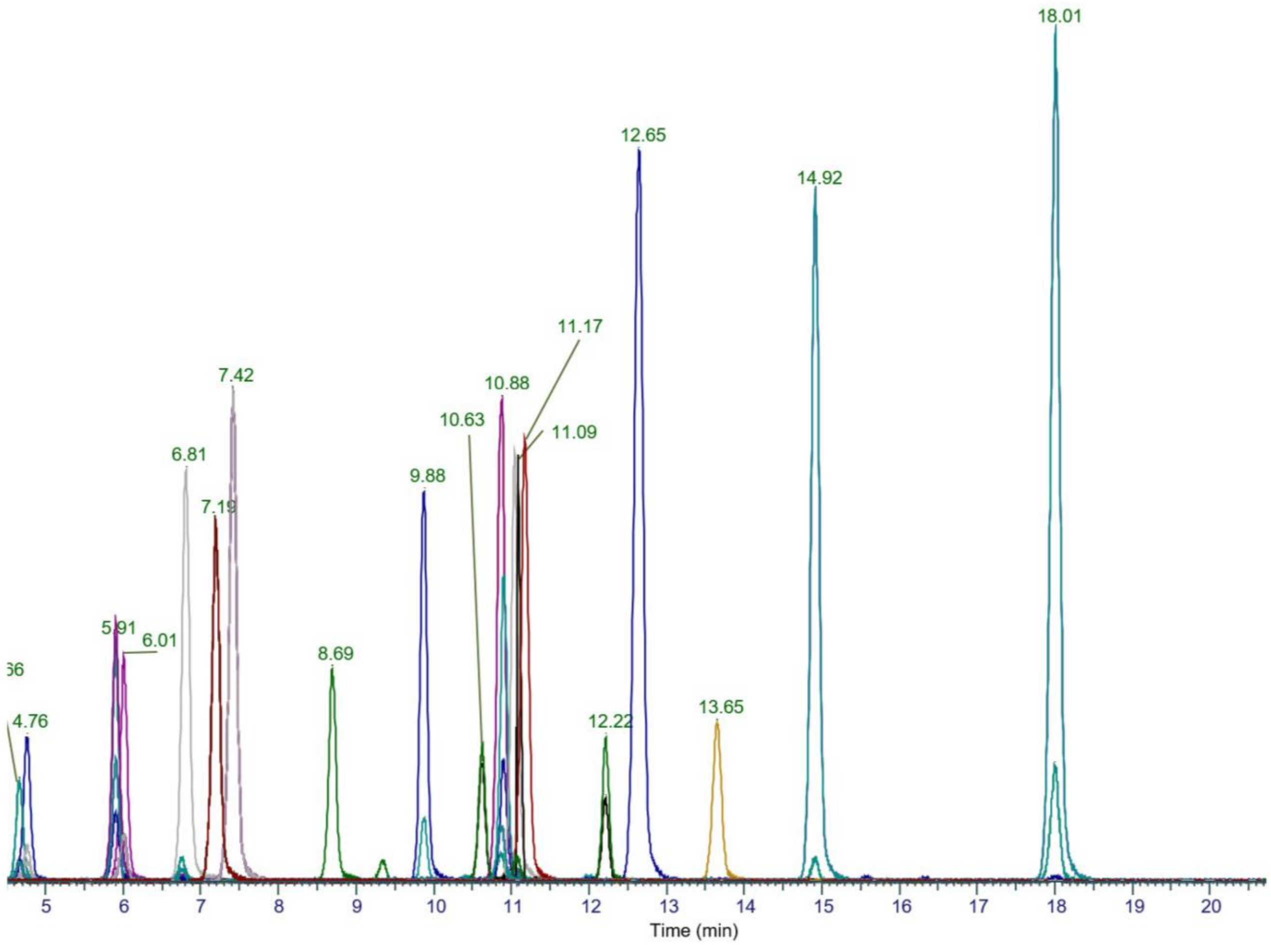

Table S1. Blood concentration (ng/mL) of cardinal ecdysteroids (**1**, **5**, **6**, **8** and **12**) in selected birds that had tick infestation. BLOQ: below limit of quantification.

No.	BIRD SP.	DATE	DAY/YEAR	apolytic ticks / all (%)	20E (1)	2-Deoxy-20E (5)	Ajugasterone C (6)	Dacryhainansterone (8)	Polypodine B (12)
S03	PRU MOD	03.21	79	0/5	147.6	0.0	0.0	43.3	BLOQ
S09	FRI COE	03.21	79	0/1	88.2	117.2	87.6	108.5	0.0
S15	ERI RUB	03.21	79	0/1	167.0	73.2	120.2	140.0	0.0
S16	ERI RUB	03.21	79	0/5	26.4	15.5	24.3	44.0	0.0
S18	TUR PHI	03.21	79	1/2 (50%)	56.0	28.5	34.6	20.8	BLOQ
S19	PRU MOD	03.22	80	0/1	BLOQ	5.7	BLOQ	7.6	0.0
S21	TUR MER	03.22	80	0/2	3.6	2.7	4.3	0.0	0.0
S26	PRU MOD	03.22	80	0/7	0.0	0.0	0.0	0.0	0.0
S28	PRU MOD	03.22	80	0/1	22.8	11.6	21.5	9.4	0.0
S29	PRU MOD	03.22	80	0/1	BLOQ	0.0	3.6	0.0	0.0
S30	COC COC	03.22	80	2/3 (66%)	105.9	BLOQ	82.3	84.2	0.0
S32	FRI COE	03.22	80	0/1	293.5	0.0	269.4	119.9	0.0
S33	ERI RUB	03.22	80	0/1	41.4	BLOQ	31.7	0.0	0.0
S34	PRU MOD	03.22	80	0/1	89.6	BLOQ	39.1	34.0	0.0
S35	COC COC	03.22	80	0/1	30.6	0.0	0.0	0.0	0.0
S36	COC COC	03.22	80	0/1	676.5	BLOQ	94.9	136.6	0.0
S37	PRU MOD	03.22	80	0/3	343.2	106.6	BLOQ	168.6	0.0
S41	TRO TRO	03.29	87	0/1	BLOQ	85.7	137.9	190.1	0.0
S45	TUR MER	03.29	87	0/6	165.9	67.1	79.4	84.0	0.0
S50	ERI RUB	03.29	87	0/1	0.0	0.0	0.0	0.0	0.0
S62	PRU MOD	03.29	87	0/3	322.2	119.6	0.0	171.6	BLOQ
S64	ERI RUB	03.29	87	0/1	39.2	0.0	0.0	517.1	0.0
S65	ERI RUB	03.29	87	0/1	235.4	116.9	186.0	1211.8	0.0
S75	ERI RUB	04.07	96	1/2 (50%)	82.7	36.3	0.0	321.0	0.0
S76	PRU MOD	04.07	96	0/15	490.1	164.2	171.9	208.1	0.0
S77	ERI RUB	04.07	96	0/3	BLOQ	0.0	0.0	433.7	0.0
S78	ERI RUB	04.08	97	0/16	93.4	44.2	47.7	0.0	0.0
S79	COC COC	04.08	97	0/2	0.0	0.0	0.0	459.0	0.0
S80	TUR MER	04.09	98	0/14	0.0	0.0	0.0	0.0	0.0
U003	TUR MER	04.18	107	0/3	4.1	0.0	0.0	0.0	0.0
U013	SYL ATR	04.19	108	0/1	0.0	0.0	0.0	11.3	0.0
U017	TUR MER	04.19	108	0/4	0.0	0.0	0.0	18.2	0.0
U018	TUR MER	04.19	108	0/7	43.7	0.0	0.0	1316.1	0.0
U035	SYL ATR	04.19	108	0/2	148.9	BLOQ	0.0	33.2	0.0
U036	LUS MEG	05.20	139	0/5	28.0	0.0	0.0	51.8	0.0
U052	ACR SCI	05.30	149	1/2 (50%)	198.0	84.9	98.3	94.9	0.0
U054	TUR PHI	05.30	149	0/2	848.1	363.0	421.3	335.5	0.0
U058	LOC LUS	07.07	187	3/3 (100%)	75.0	BLOQ	0.0	48.5	0.0
U061	LOC LUS	07.07	187	1/6 (17%)	1011.4	399.4	529.4	302.1	0.0
U065	ACR SCH	07.07	187	0/1	188.9	71.1	0.0	85.7	0.0
U066	ACR SCH	07.07	187	2/4 (50%)	3043.5	60.6	0.0	150.1	0.0
U067	LOC LUS	07.07	187	1/1 (100%)	194.5	0.0	0.0	110.7	0.0
U069	LOC LUS	07.07	187	2/3 (67%)	0.0	0.0	0.0	83.9	0.0
U072	SYL ATR	08.03	215	1/2 (50%)	36.8	59.4	61.2	211.0	BLOQ
U076	SYL ATR	08.03	215	1/2 (50%)	316.9	373.8	396.7	1190.4	BLOQ
U077	TUR PHI	08.03	215	2/3 (67%)	81.7	BLOQ	0.0	0.0	BLOQ
U081	ACR PAL	08.04	216	1/1 (100%)	369.8	862.1	870.2	3581.8	BLOQ
U086	ERI RUB	08.05	217	1/17 (6%)	129.0	242.0	242.4	924.3	76.5
U087	LUS MEG	08.04	216	0/1	103.3	75.6	80.3	302.7	34.1
U088	LOC LUS	08.05	217	5/18 (28%)	694.2	1038.4	981.5	2668.0	433.2
U090	ACR SCH	08.05	217	2/2 (100%)	43.1	26.0	28.4	50.0	BLOQ
U091	TUR MER	08.05	217	7/7 (100%)	1215.5	499.4	567.1	382.8	0.0
U092	LOC LUS	08.05	217	2/2 (100%)	469.9	357.5	396.7	887.9	BLOQ
U093	ACR SCI	08.05	217	1/2 (50%)	633.9	1059.9	1122.9	2867.0	BLOQ
U094	ACR SCI	08.05	217	0/1	289.8	462.2	546.0	2122.5	BLOQ
U095	ERI RUB	08.05	217	1/6 (17%)	267.9	281.3	326.9	444.6	204.4
U098	ERI RUB	08.05	217	1/2 (50%)	50.9	19.9	23.9	0.0	0.0
U102	ACR PAL	08.06	218	1/6 (17%)	27.3	0.0	0.0	0.0	0.0
U103	ACR SCI	08.06	218	1/1 (100%)	21.0	0.0	0.0	0.0	0.0
U104	LUS MEG	08.06	218	1/2 (50%)	22.5	0.0	0.0	0.0	0.0
U109	LOC LUS	08.06	218	0/6	68.2	BLOQ	0.0	0.0	0.0
U110	TUR MER	08.06	218	0/1	12.6	0.0	0.0	0.0	0.0

U113	ACR SCI	08.07	219	0/22	25.6	0.0	0.0	53.1	0.0
U114	ERI RUB	08.07	219	0/1	147.3	62.4	80.5	0.0	0.0
U116	ACR SCH	08.07	219	1/1 (100%)	7.7	0.0	0.0	0.0	0.0
U117	SYL ATR	08.07	219	1/1 (100%)	18.8	9.3	10.1	0.0	0.0
U118	ERI RUB	08.07	219	0/1	16.0	0.0	0.0	0.0	0.0
U120	LOC LUS	08.07	219	1/10 (10%)	107.3	56.7	74.6	1755.9	0.0
U121	LUS MEG	08.08	220	1/6 (17%)	341.7	130.4	157.3	288.3	0.0
U122	ERI RUB	08.08	220	1/4 (25%)	62.0	0.0	0.0	1405.1	0.0
U123	SYL ATR	08.08	220	1/3 (33%)	130.7	BLOQ	0.0	790.2	0.0
U124	SYL ATR	08.08	220	2/3 (67%)	0.0	0.0	0.0	373.9	0.0
U125	LUS MEG	08.08	220	1/1 (100%)	102.5	0.0	0.0	1645.9	0.0
U126	ERI RUB	08.08	220	0/3	34.1	14.2	15.6	26.0	0.0
U127	ERI RUB	08.08	220	0/2	0.0	0.0	0.0	720.5	0.0
U128	LOC LUS	08.08	220	10/10 (100%)	37.6	14.3	16.8	30.8	0.0
U129	LUS MEG	08.08	220	1/1 (100%)	18.6	0.0	0.0	93.9	0.0
U130	PAR MAJ	08.08	220	0/3	40.4	BLOQ	0.0	0.0	0.0
U131	ERI RUB	08.08	220	1/2 (50%)	219.4	58.1	0.0	701.5	0.0
U132	SYL COM	08.08	220	0/1	118.7	0.0	0.0	310.1	0.0
U134	ACR SCH	08.09	221	0/1	84.1	0.0	0.0	114.6	0.0
U135	LOC LUS	08.09	221	0/1	348.7	168.7	203.1	452.4	0.0
U136	LUS MEG	08.10	222	0/1	0.0	0.0	0.0	127.9	0.0
U137	LUS LUS	08.10	222	2/2 (100%)	101.4	0.0	0.0	239.5	0.0
U138	ERI RUB	08.17	228	0/4	0.0	0.0	0.0	78.8	0.0
U139	ACR SCI	08.17	228	0/1	233.0	115.6	138.3	358.3	0.0
U140	ACR SCI	08.17	228	0/1	32.0	0.0	19.5	115.6	0.0
U141	ACR SCH	08.17	228	1/1 (100%)	141.3	69.0	104.5	1266.6	0.0
U142	ACR SCI	08.18	229	1/1 (100%)	123.4	60.7	76.8	507.4	0.0
U143	ACR SCI	08.18	229	0/1	223.6	106.9	139.0	395.7	0.0
U144	ACR SCI	08.19	230	1/1 (100%)	51.2	0.0	0.0	140.2	0.0
U145	LOC LUS	08.20	231	2/2 (100%)	134.2	0.0	0.0	113.0	0.0
U146	LUS MEG	08.21	232	6/13 (46%)	120.3	52.3	61.4	114.1	0.0
U147	LOC LUS	08.22	233	2/3 (67%)	258.3	BLOQ	0.0	0.0	0.0
U148	LUS SVE	08.22	233	1/1 (100%)	49.8	BLOQ	29.5	168.0	0.0
U149	ERI RUB	08.22	233	1/3 (33%)	120.1	47.0	75.7	478.3	0.0
U150	ACR SCH	08.23	234	1/1 (100%)	149.6	74.0	89.3	1032.3	0.0
U151	SYL ATR	08.23	234	1/2 (50%)	94.7	BLOQ	0.0	764.5	0.0
Ux01	SYL ATR	09.04	246	1/1 (100%)	0.0	0.0	0.0	711.3	0.0
Ux02	SYL COM	09.04	246	1/1 (100%)	333.1	93.6	126.5	498.2	0.0
Ux03	SYL COM	09.04	246	0/1	61.4	0.0	0.0	605.3	0.0
Ux04	SYL COM	09.04	246	1/1 (100%)	59.9	0.0	0.0	348.3	0.0
Ux05	ERI RUB	09.04	246	3/6 (50%)	193.9	82.5	99.4	327.3	0.0
Ux06	TUR MER	09.04	246	4/9 (44%)	19.8	0.0	0.0	185.8	0.0
Ux07	PAR MAJ	09.04	246	0/1	55.3	27.9	0.0	273.8	0.0
Ux08	SYL ATR	09.05	247	0/3	BLOQ	0.0	0.0	349.9	0.0
Ux09	ERI RUB	08.23	234	0/4	166.3	0.0	0.0	273.9	0.0
Ux10	ERI RUB	09.05	247	5/6 (83%)	36.5	13.7	23.0	58.3	0.0
Ux11	ERI RUB	09.05	247	2/4 (50%)	75.9	0.0	0.0	43.2	0.0
Ux12	ERI RUB	09.05	247	3/5 (60%)	402.4	0.0	0.0	114.3	0.0
Ux13	TUR PHI	09.05	247	0/1	11.6	0.0	0.0	13.3	0.0
Ux14	SYL COM	09.06	248	1/1 (100%)	76.2	33.4	40.1	122.7	0.0
Ux15	ERI RUB	09.07	249	1/4 (25%)	144.3	0.0	0.0	126.6	0.0
Ux16	ERI RUB	09.07	249	2/3 (67%)	67.9	0.0	0.0	173.3	0.0
Ux17	ERI RUB	09.07	249	2/4 (50%)	94.5	0.0	0.0	140.9	0.0

Table S2. The list of the compounds and the ions chosen for the data acquisition. The order of the ions follows the ion intensity ratios, compounds are listed in the order of their retention times.

**Compound ID**	**m/z 1 (Da)**	**m/z 2 (Da)**	**m/z 3 (Da)**	**m/z 4 (Da)**
11α-Hydroxypoststerone (**20**)	379.21152			
Rubrosterone (**14**)	335.1853			
3-epi-20E (**25**)	445.29496	463.30539	481.31598	
Herkesterone (**24**)	495.29524	459.27438	477.28516	
20-Hydroxyecdysone (20E; **1**)	481.31598	445.29504	463.30557	
Polypodine B (**12**)	497.31089	461.29037	479.30099	
5α-20E (**17**)	445.29517	427.28448	463.30545	481.31598
9,11-Didehydropoststerone (**10**)	361.20095			
5β-Hydroxypoststerone (**19**)	379.21152			
Poststerone (**13**)	363.2166			
Cyasterone (**23**)	521.31089			
Ecdysone (**9**)	447.31094	429.30078	465.32107	
20E 2-acetate (**2**)	523.32654			
Ajugasterone C (**6**)	481.31598	465.32169	429.3006	447.3111
2-Deoxy-20E (**5**)	465.32107	429.30057	447.31119	
Calonysterone (**7**)	477.28468	459.27393	441.26343	
20E 22-acetate (**16**)	523.32654	505.31589		
Ajugalactone (**22**)	517.27959	499.26984		
20E 3-acetate (**3**)	487.30569	523.32654	469.29532	487.30569
2-Deoxypoststerone (**11**)	347.22169			
Compound **21**	359.1853			
Dacryhainansterone (**8**)	463.30563			
Shidasterone (**15**)	463.30542	445.29605		
20E 20,22-acetonide (**4**)	521.34728	503.33719		

Table S3. Summary of the Calibration samples and their concentrations in the working standard (Stock) in the order of their retention time (RT). Calibration level concentrations are shown in decreasing order (level 5 to 1, L5 to L1, respectively) in ng/ml.

			Stock (µg/ml)	Concentration of the calibration levels (ng/ml)				
#	Compound	RT (min)		L5	L4	L3	L2	L1
1	11α-Hydroxypoststerone (**20**)	3.13	65.22	72.46	24.16	8.05	2.68	0.90
2	Rubrosterone (**14**)	3.87	66.09	73.43	24.48	8.16	2.72	0.91
3	3-epi-20E (**25**)	4.68	43.04	47.83	15.94	5.31	1.77	0.59
4	Herkesterone (**24**)	4.76	60.87	67.63	22.54	7.52	2.51	0.84
5	20-Hydroxyecdysone (20E; **1**)	5.92	31.06	34.51	11.50	3.83	1.28	0.43
6	Polypodine B (**12**)	6.02	31.06	34.51	11.50	3.83	1.28	0.43
7	5α-20E (**17**)	6.78	4.35	4.83	1.61	0.54	0.18	0.06
8	9,11-Didehydropoststerone (**10**)	6.82	44.78	49.76	16.59	5.53	1.84	0.61
9	5β-Hydroxypoststerone (**19**)	7.21	52.17	57.97	19.32	6.44	2.15	0.72
10	Poststerone (**13**)	7.44	31.06	34.51	11.50	3.83	1.28	0.43
11	Cyasterone (**23**)	8.70	66.96	74.40	24.80	8.27	2.76	0.92
12	Ecdysone (**9**)	9.89	34.16	37.96	12.65	4.22	1.41	0.47
13	20E 2-acetate (**2**)	10.63	47.83[a]	53.14	17.71	5.90	1.97	0.66
14	Ajugasterone C (**6**)	10.88	34.16	37.96	12.65	4.22	1.41	0.47
15	2-Deoxy-20E (**5**)	10.92	31.06	34.51	11.50	3.83	1.28	0.43
16	Calonysterone (**7**)	11.05	50.43	56.04	18.68	6.23	2.08	0.69
17	20E 22-acetate (**16**)	11.08	46.09	51.21	17.07	5.69	1.90	0.63
18	Ajugalactone (**22**)	11.20	50.43	56.04	18.68	6.23	2.08	0.69
19	20E 3-acetate (**3**)	12.23	47.83[a]	53.14	17.71	5.90	1.97	0.66
20	2-Deoxypoststerone (**11**)	12.65	41.30	45.89	15.30	5.10	1.70	0.57
21	Compound **21**	13.66	2.17	2.42	0.81	0.27	0.09	0.03
22	Dacryhainansterone (**8**)	14.93	34.16	37.96	12.65	4.22	1.41	0.47
23	Shidasterone (**15**)	18.04	43.91	48.79	16.26	5.42	1.81	0.60
24	20E 20,22-acetonide (**4**)	23.35	50.00	55.56	18.52	6.17	2.06	0.69

[a] Calibration for compounds **2** and **3** was performed from a stock solution containing their mixture in a ratio of 0.23642 : 0.76358 (SD= 0.010586) at a total concentration of 47.83 µg/mL.

Table S4. Summary of the calibration curve fitting.

Compound ID	Curve Type	y-Intercept	Slope	R^2
11α-Hydroxypoststerone (**20**)	Linear	-3.90E+04	4.18E+05	0.9996
Rubrosterone (**14**)	Linear	-1.24E+04	4.97E+05	0.9987
3-epi-20E (**25**)	Linear	-1.79E+04	1.23E+05	0.9977
Herkesterone (**24**)	Linear	-3.00E+04	1.60E+05	0.9978
20-Hydroxyecdysone (20E; **1**)	Linear	-1.31E+05	9.87E+05	0.9966
Polypodine B (**12**)	Linear	-7.57E+04	6.80E+05	0.9978
5α-20E (**17**)	Linear	-2.68E+04	5.82E+05	0.9979
9,11-Didehydropoststerone (**10**)	Linear	3.33E+04	4.65E+05	0.9988
5β-Hydroxypoststerone (**19**)	Linear	-3.14E+04	3.32E+05	0.9981
Poststerone (**13**)	Linear	5.01E+05	7.62E+05	0.9975
Cyasterone (**23**)	Linear	-1.18E+04	1.40E+05	0.9962
Ecdysone (**9**)	Linear	1.37E+04	6.90E+05	0.9974
20E 2-acetate (**2**)	Linear	3.60E+03	8.43E+04	0.9953
Ajugasterone C (**6**)	Linear	-1.36E+05	1.54E+06	0.9968
2-Deoxy-20E (**5**)	Linear	-1.04E+05	9.10E+05	0.9963
Calonysterone (**7**)	Linear	-1.25E+05	9.11E+05	0.9969
20E 22-acetate (**16**)	Linear	-2.88E+05	8.23E+05	0.9912
Ajugalactone (**22**)	Linear	-3.04E+04	4.73E+05	0.9963
20E 3-acetate (**3**)	Linear	7.18E+02	2.77E+05	0.9958
2-Deoxypoststerone (**11**)	Linear	2.99E+04	9.70E+05	0.9977
Compound **21**	Linear	-3.20E+03	3.75E+06	0.998
Dacryhainansterone (**8**)	Linear	-2.66E+04	1.00E+06	0.9977
Shidasterone (**15**)	Linear	1.97E+04	1.34E+06	0.9994
20E 20,22-acetonide (**4**)	Linear	-1.09E+03	7.52E+05	0.9983

Table S5. The complete data set for the blood ecdysteroid concentrations (ng/ml) for all birds. BLOQ: below limit of quantification.

No.	BIRD SP.	DATE	DAY/YEAR	20E (1)	20E 2-acetate (2)	20E 3-acetate (3)	20E 20,22-acetonide (4)	2-Deoxy-20E (5)	Ajugasterone C (6)	Calonysterone (7)	Dacryhainansterone (8)	Ecdysone (9)	9,11-Didehydro-poststerone (10)	2-Deoxy-poststerone (11)	Polypodine B (12)	Poststerone (13)	Rubrosterone (14)	Shidasterone (15)
S02	ERI RUB	03.21	79	431.0	404.8	1350.9	89.9	0.0	0.0	315.4	57.3	0.0	BLOQ	0.0	BLOQ	153.5	31.8	59.9
S03	PRU MOD	03.21	79	147.6	31.8	BLOQ	102.9	0.0	0.0	141.7	43.3	0.0	BLOQ	0.0	BLOQ	35.5	BLOQ	47.8
S04	TRO TRO	03.21	79	37.6	16.3	75.1	BLOQ	0.0	0.0	30.3	67.2	0.0	BLOQ	0.0	0.0	7.7	31.4	14.7
S06	ERI RUB	03.21	79	173.4	41.2	BLOQ	BLOQ	0.0	0.0	211.5	82.0	0.0	35.4	0.0	BLOQ	246.9	BLOQ	174.1
S07	ERI RUB	03.21	79	210.8	90.9	286.4	202.1	733.7	452.0	686.3	171.7	0.0	135.5	0.0	BLOQ	960.7	BLOQ	1214.5
S08	ERI RUB	03.21	79	74.6	BLOQ	BLOQ	BLOQ	176.5	114.5	158.2	59.9	0.0	57.0	0.0	BLOQ	243.4	0.0	338.7
S09	FRI COE	03.21	79	88.2	27.2	BLOQ	75.5	117.2	87.6	209.4	108.5	0.0	BLOQ	0.0	0.0	164.7	BLOQ	623.4
S11	ERI RUB	03.21	79	94.4	BLOQ	BLOQ	52.9	53.9	55.2	99.0	49.8	0.0	BLOQ	0.0	0.0	BLOQ	0.0	57.5
S12	ERI RUB	03.21	79	197.9	BLOQ	BLOQ	BLOQ	82.7	90.1	137.3	98.4	0.0	BLOQ	0.0	0.0	BLOQ	0.0	215.7
S13	TUR PHI	03.21	79	12.9	BLOQ	BLOQ	BLOQ	8.4	BLOQ	12.3	9.3	0.0	BLOQ	0.0	0.0	7.1	BLOQ	11.6
S15	ERI RUB	03.21	79	167.0	36.3	BLOQ	147.5	73.2	120.2	143.4	140.0	BLOQ	BLOQ	0.0	0.0	BLOQ	BLOQ	249.5
S16	ERI RUB	03.21	79	26.4	BLOQ	BLOQ	25.4	15.5	24.3	23.0	44.0	0.0	BLOQ	0.0	0.0	BLOQ	0.0	53.2
S17	ERI RUB	03.21	79	70.7	15.4	BLOQ	42.4	43.7	59.0	58.1	55.6	0.0	BLOQ	0.0	0.0	BLOQ	BLOQ	73.8
S18	TUR PHI	03.21	79	56.0	13.4	BLOQ	16.1	28.5	34.6	21.3	20.8	0.0	16.2	0.0	BLOQ	37.1	BLOQ	31.5
S19	PRU MOD	03.22	80	BLOQ	BLOQ	BLOQ	BLOQ	5.7	BLOQ	BLOQ	7.6	0.0	BLOQ	0.0	0.0	0.0	0.0	14.9
S21	TUR MER	03.22	80	3.6	BLOQ	BLOQ	BLOQ	2.7	4.3	BLOQ	0.0	0.0	BLOQ	0.0	0.0	3.4	0.0	BLOQ
S22	ERI RUB	03.22	80	44.6	BLOQ	27.3	BLOQ	25.5	42.2	BLOQ	19.7	0.0	BLOQ	0.0	0.0	23.8	BLOQ	BLOQ
S24	ERI RUB	03.22	80	162.0	48.1	BLOQ	BLOQ	0.0	239.1	96.6	98.1	0.0	BLOQ	0.0	0.0	BLOQ	0.0	BLOQ
S25	ERI RUB	03.22	80	0.0	BLOQ	BLOQ	0.0	0.0	7.1	BLOQ	0.0	0.0	BLOQ	0.0	0.0	BLOQ	0.0	0.0
S26	PRU MOD	03.22	80	0.0	BLOQ	BLOQ	BLOQ	0.0	0.0	BLOQ	0.0	0.0	0.0	0.0	0.0	BLOQ	0.0	0.0
S27	PRU MOD	03.22	80	123.6	20.2	BLOQ	26.8	0.0	176.7	51.9	35.1	0.0	BLOQ	0.0	0.0	30.9	BLOQ	24.6
S28	PRU MOD	03.22	80	22.8	BLOQ	BLOQ	BLOQ	11.6	21.5	8.1	9.4	0.0	BLOQ	0.0	0.0	BLOQ	BLOQ	BLOQ
S29	PRU MOD	03.22	80	BLOQ	BLOQ	BLOQ	BLOQ	0.0	3.6	BLOQ	0.0	0.0	0.0	0.0	0.0	BLOQ	0.0	BLOQ
S30	COC COC	03.22	80	105.9	BLOQ	BLOQ	BLOQ	BLOQ	82.3	BLOQ	84.2	0.0	BLOQ	0.0	0.0	BLOQ	0.0	79.3
S31	ACR SCH	03.22	80	277.2	BLOQ	BLOQ	BLOQ	BLOQ	105.4	BLOQ	90.7	0.0	0.0	0.0	0.0	BLOQ	BLOQ	BLOQ
S32	FRI COE	03.22	80	293.5	49.7	160.0	BLOQ	0.0	269.4	BLOQ	119.9	0.0	BLOQ	0.0	0.0	65.3	BLOQ	107.8
S33	ERI RUB	03.22	80	41.4	7.6	BLOQ	BLOQ	BLOQ	31.7	BLOQ	0.0	0.0	BLOQ	0.0	0.0	0.0	BLOQ	BLOQ
S34	PRU MOD	03.22	80	89.6	BLOQ	BLOQ	BLOQ	BLOQ	39.1	BLOQ	34.0	BLOQ	BLOQ	0.0	0.0	BLOQ	0.0	38.6
S35	COC COC	03.22	80	30.6	0.0	BLOQ	BLOQ	0.0	0.0	BLOQ	0.0	0.0	0.0	0.0	0.0	BLOQ	0.0	BLOQ
S36	COC COC	03.22	80	676.5	BLOQ	BLOQ	112.3	BLOQ	94.9	BLOQ	136.6	BLOQ	BLOQ	0.0	0.0	BLOQ	0.0	81.0
S37	PRU MOD	03.22	80	343.2	BLOQ	BLOQ	BLOQ	106.6	BLOQ	BLOQ	168.6	0.0	BLOQ	0.0	0.0	BLOQ	BLOQ	BLOQ
S38	ERI RUB	03.29	87	43.0	BLOQ	0.0	0.0	11.1	0.0	0.0	0.0	0.0	BLOQ	0.0	0.0	BLOQ	0.0	0.0
S39	ERI RUB	03.29	87	58.4	12.1	0.0	0.0	0.0	102.1	30.0	0.0	0.0	BLOQ	0.0	0.0	0.0	0.0	0.0
S40	ERI RUB	03.29	87	BLOQ	11.4	BLOQ	BLOQ	21.5	41.9	30.8	0.0	0.0	BLOQ	0.0	0.0	0.0	0.0	BLOQ
S41	TRO TRO	03.29	87	BLOQ	112.2	BLOQ	BLOQ	85.7	137.9	238.2	190.1	0.0	BLOQ	0.0	0.0	BLOQ	BLOQ	BLOQ
S42	PRU MOD	03.29	87	593.1	45.8	162.9	121.9	116.7	143.3	BLOQ	148.0	0.0	BLOQ	0.0	0.0	BLOQ	BLOQ	BLOQ
S43	ERI RUB	03.29	87	0.0	0.0	BLOQ	BLOQ	0.0	0.0	BLOQ	0.0	0.0	BLOQ	0.0	0.0	BLOQ	0.0	0.0
S44	PRU MOD	03.29	87	25.3	BLOQ	BLOQ	BLOQ	BLOQ	16.5	BLOQ	18.4	0.0	BLOQ	0.0	0.0	BLOQ	BLOQ	BLOQ
S45	TUR MER	03.29	87	165.9	24.6	BLOQ	BLOQ	67.1	79.4	BLOQ	84.0	BLOQ	BLOQ	0.0	0.0	65.9	BLOQ	BLOQ
S46	PRU MOD	03.29	87	197.9	BLOQ	BLOQ	BLOQ	BLOQ	0.0	BLOQ	126.3	0.0	BLOQ	0.0	0.0	BLOQ	0.0	BLOQ
S47	ERI RUB	03.29	87	281.1	50.1	157.7	132.3	106.8	126.4	BLOQ	149.1	0.0	BLOQ	0.0	0.0	BLOQ	BLOQ	BLOQ
S48	ERI RUB	03.29	87	31.8	BLOQ	BLOQ	BLOQ	12.4	15.3	BLOQ	24.3	0.0	BLOQ	0.0	0.0	BLOQ	BLOQ	BLOQ
S49	ERI RUB	03.29	87	33.8	BLOQ	BLOQ	BLOQ	0.0	0.0	BLOQ	24.9	0.0	0.0	0.0	0.0	0.0	0.0	BLOQ
S50	ERI RUB	03.29	87	0.0	0.0	0.0	BLOQ	0.0	0.0	0.0	0.0	0.0	0.0	0.0	0.0	0.0	0.0	0.0
S51	ERI RUB	03.29	87	34.0	BLOQ	BLOQ	BLOQ	0.0	0.0	BLOQ	0.0	0.0	BLOQ	0.0	0.0	0.0	0.0	BLOQ
S52	ERI RUB	03.29	87	BLOQ	BLOQ	BLOQ	0.0	0.0	0.0	BLOQ	0.0	0.0	0.0	0.0	0.0	BLOQ	0.0	0.0
S53	ERI RUB	03.29	87	643.6	59.8	199.0	BLOQ	110.4	130.0	BLOQ	155.1	0.0	BLOQ	0.0	0.0	BLOQ	BLOQ	BLOQ
S54	TUR MER	03.29	87	16.4	BLOQ	BLOQ	BLOQ	0.0	0.0	BLOQ	8.1	0.0	0.0	0.0	0.0	BLOQ	BLOQ	0.0
S55	ERI RUB	03.29	87	5.7	0.0	0.0	BLOQ	0.0	0.0	0.0	0.0	0.0	0.0	0.0	0.0	0.0	0.0	0.0
S56	ERI RUB	03.29	87	18.4	BLOQ	BLOQ	BLOQ	0.0	0.0	BLOQ	0.0	0.0	0.0	0.0	0.0	BLOQ	0.0	0.0
S57	ERI RUB	03.29	87	0.0	BLOQ	BLOQ	0.0	0.0	0.0	BLOQ	0.0	0.0	0.0	0.0	0.0	BLOQ	BLOQ	0.0
S58	ERI RUB	03.29	87	67.4	BLOQ	31.4	BLOQ	36.9	41.2	BLOQ	30.7	0.0	BLOQ	0.0	0.0	0.0	BLOQ	BLOQ
S59	ERI RUB	03.29	87	818.9	BLOQ	BLOQ	BLOQ	174.2	154.0	BLOQ	141.6	0.0	141.4	0.0	0.0	401.1	BLOQ	BLOQ
S60	ERI RUB	03.29	87	16.4	BLOQ	BLOQ	BLOQ	BLOQ	7.8	28.4	110.4	0.0	BLOQ	0.0	0.0	BLOQ	BLOQ	BLOQ
S61	TUR PHI	03.29	87	124.2	BLOQ	BLOQ	BLOQ	0.0	0.0	0.0	1998.9	0.0	184.4	0.0	0.0	BLOQ	0.0	0.0

S62	PRU MOD	03.29	87	322.2	BLOQ	BLOQ	BLOQ	119.6	0.0	BLOQ	171.6	BLOQ	BLOQ	0.0	BLOQ	96.7	BLOQ	0.0
S63	ERI RUB	03.29	87	22.4	BLOQ	BLOQ	BLOQ	7.6	8.8	11.6	103.9	0.0	14.7	0.0	0.0	BLOQ	BLOQ	BLOQ
S64	ERI RUB	03.29	87	39.2	BLOQ	BLOQ	BLOQ	0.0	0.0	38.6	517.1	0.0	BLOQ	0.0	0.0	BLOQ	0.0	0.0
S65	ERI RUB	03.29	87	235.4	BLOQ	BLOQ	BLOQ	116.9	186.0	BLOQ	1211.8	0.0	206.2	0.0	0.0	BLOQ	BLOQ	BLOQ
S66	PRU MOD	03.29	87	431.6	64.0	BLOQ	185.4	201.5	227.2	BLOQ	1302.3	0.0	BLOQ	0.0	0.0	BLOQ	BLOQ	BLOQ
S67	ERI RUB	03.29	87	58.1	0.0	0.0	0.0	BLOQ	28.8	BLOQ	0.0	0.0	BLOQ	0.0	0.0	BLOQ	BLOQ	0.0
S68	TUR MER	03.29	87	0.0	0.0	0.0	0.0	0.0	0.0	0.0	0.0	0.0	0.0	0.0	0.0	BLOQ	0.0	0.0
S69	ERI RUB	03.29	87	736.3	116.5	369.2	BLOQ	301.1	356.7	BLOQ	2603.1	0.0	431.6	0.0	0.0	BLOQ	BLOQ	BLOQ
S70	ERI RUB	03.29	87	0.0	0.0	0.0	0.0	0.0	0.0	BLOQ	41.3	0.0	BLOQ	0.0	0.0	0.0	0.0	0.0
S71	PRU MOD	03.29	87	481.7	BLOQ	BLOQ	BLOQ	89.8	104.0	152.4	199.2	0.0	BLOQ	0.0	0.0	108.3	BLOQ	0.0
S72	ERI RUB	03.29	87	797.7	BLOQ	BLOQ	BLOQ	242.3	292.2	BLOQ	437.8	0.0	BLOQ	0.0	0.0	BLOQ	BLOQ	0.0
S73	ERI RUB	03.29	87	318.4	BLOQ	BLOQ	BLOQ	92.4	0.0	148.3	623.2	0.0	BLOQ	0.0	0.0	BLOQ	BLOQ	BLOQ
S74	ERI RUB	03.29	87	162.6	BLOQ	BLOQ	48.9	42.2	45.5	76.4	240.6	0.0	BLOQ	0.0	0.0	0.0	0.0	BLOQ
S75	ERI RUB	04.07	96	82.7	BLOQ	BLOQ	BLOQ	36.3	0.0	BLOQ	321.0	0.0	77.5	0.0	0.0	0.0	BLOQ	BLOQ
S76	PRU MOD	04.07	96	490.1	BLOQ	BLOQ	BLOQ	164.2	171.9	BLOQ	208.1	BLOQ	0.0	0.0	0.0	148.4	BLOQ	BLOQ
S77	ERI RUB	04.07	96	BLOQ	BLOQ	BLOQ	BLOQ	0.0	0.0	BLOQ	433.7	0.0	BLOQ	0.0	0.0	0.0	0.0	0.0
S78	ERI RUB	04.08	97	93.4	BLOQ	BLOQ	BLOQ	44.2	47.7	BLOQ	0.0	0.0	BLOQ	0.0	0.0	BLOQ	BLOQ	0.0
S79	COC COC	04.08	97	0.0	BLOQ	BLOQ	0.0	0.0	0.0	BLOQ	459.0	0.0	BLOQ	0.0	0.0	0.0	0.0	0.0
S80	TUR MER	04.09	98	0.0	0.0	0.0	0.0	0.0	0.0	0.0	0.0	0.0	0.0	0.0	0.0	BLOQ	0.0	0.0
U001	ERI RUB	04.18	107	37.0	BLOQ	BLOQ	BLOQ	BLOQ	0.0	15.8	47.7	0.0	BLOQ	0.0	0.0	10.0	BLOQ	0.0
U002	ERI RUB	04.18	107	2.6	BLOQ	BLOQ	BLOQ	2.1	0.0	BLOQ	3.7	0.0	BLOQ	0.0	0.0	5.8	0.0	BLOQ
U003	TUR MER	04.18	107	4.1	4.4	0.0	0.0	0.0	0.0	BLOQ	0.0	0.0	BLOQ	0.0	0.0	BLOQ	0.0	0.0
U004	SYL ATR	04.18	107	6.9	BLOQ	BLOQ	BLOQ	4.4	0.0	BLOQ	126.7	0.0	9.7	0.0	0.0	BLOQ	BLOQ	BLOQ
U005	SYL ATR	04.18	107	196.4	BLOQ	BLOQ	BLOQ	78.5	0.0	BLOQ	860.4	0.0	BLOQ	0.0	0.0	BLOQ	BLOQ	BLOQ
U006	ACR SCI	04.18	107	670.5	BLOQ	BLOQ	BLOQ	0.0	0.0	BLOQ	1642.3	0.0	BLOQ	0.0	0.0	916.9	BLOQ	BLOQ
U007	SYL ATR	04.18	107	963.7	55.1	190.2	BLOQ	0.0	128.6	BLOQ	668.7	0.0	BLOQ	0.0	0.0	91.2	BLOQ	BLOQ
U008	SYL ATR	04.18	107	14.2	BLOQ	BLOQ	BLOQ	9.0	9.4	BLOQ	247.7	0.0	26.4	0.0	0.0	11.1	0.0	BLOQ
U009	CAR CHL	04.18	107	25.3	BLOQ	BLOQ	BLOQ	0.0	0.0	0.0	306.2	0.0	19.5	0.0	0.0	BLOQ	0.0	BLOQ
U010	SYL ATR	04.18	107	15.8	0.0	BLOQ	BLOQ	0.0	0.0	0.0	275.5	0.0	20.0	0.0	0.0	BLOQ	BLOQ	BLOQ
U011	SYL ATR	04.19	108	26.2	BLOQ	BLOQ	BLOQ	0.0	0.0	BLOQ	32.8	0.0	BLOQ	0.0	0.0	BLOQ	0.0	0.0
U012	ERI RUB	04.19	108	0.0	0.0	BLOQ	BLOQ	0.0	0.0	BLOQ	5.9	0.0	BLOQ	0.0	0.0	BLOQ	0.0	0.0
U013	SYL ATR	04.19	108	0.0	BLOQ	BLOQ	BLOQ	0.0	0.0	BLOQ	11.3	0.0	BLOQ	0.0	0.0	BLOQ	0.0	0.0
U014	LUS MEG	04.19	108	273.8	20.9	BLOQ	BLOQ	0.0	48.2	BLOQ	46.8	0.0	BLOQ	0.0	0.0	BLOQ	BLOQ	BLOQ
U015	SYL ATR	04.19	108	9.1	BLOQ	BLOQ	BLOQ	0.0	0.0	BLOQ	12.5	0.0	BLOQ	0.0	0.0	0.0	BLOQ	0.0
U016	LUS MEG	04.19	108	798.5	43.4	BLOQ	BLOQ	117.8	145.3	BLOQ	203.1	0.0	BLOQ	0.0	0.0	BLOQ	BLOQ	0.0
U017	TUR MER	04.19	108	0.0	0.0	0.0	0.0	0.0	0.0	BLOQ	18.2	0.0	BLOQ	0.0	0.0	BLOQ	0.0	0.0
U018	TUR MER	04.19	108	43.7	BLOQ	BLOQ	BLOQ	0.0	0.0	BLOQ	1316.1	0.0	54.9	0.0	0.0	BLOQ	BLOQ	0.0
U019	SYL ATR	04.19	108	0.0	BLOQ	BLOQ	BLOQ	0.0	0.0	39.3	1273.7	0.0	67.5	0.0	0.0	BLOQ	0.0	0.0
U020	SYL ATR	04.19	108	56.0	BLOQ	BLOQ	BLOQ	BLOQ	0.0	BLOQ	500.3	0.0	BLOQ	0.0	0.0	BLOQ	BLOQ	0.0
U022	SYL ATR	04.19	108	44.9	10.5	36.5	BLOQ	25.2	47.3	BLOQ	25.0	0.0	BLOQ	0.0	0.0	BLOQ	BLOQ	BLOQ
U023	ERI RUB	04.19	108	BLOQ	0.0	BLOQ	BLOQ	0.0	0.0	BLOQ	184.5	0.0	0.0	0.0	0.0	BLOQ	BLOQ	0.0
U024	SYL ATR	04.19	108	150.2	BLOQ	BLOQ	BLOQ	0.0	0.0	69.2	58.0	0.0	BLOQ	0.0	0.0	38.5	0.0	0.0
U025	SYL ATR	04.19	108	190.9	22.2	83.6	BLOQ	63.1	74.0	BLOQ	119.4	0.0	BLOQ	0.0	0.0	BLOQ	BLOQ	BLOQ
U026	ACR SCI	04.19	108	46.8	BLOQ	BLOQ	BLOQ	0.0	0.0	BLOQ	117.2	0.0	BLOQ	0.0	0.0	BLOQ	0.0	0.0
U027	SYL ATR	04.19	108	60.6	10.0	BLOQ	BLOQ	BLOQ	0.0	BLOQ	61.1	0.0	BLOQ	0.0	0.0	BLOQ	BLOQ	BLOQ
U028	ACR SCI	04.19	108	392.6	66.6	227.8	BLOQ	187.2	204.4	BLOQ	403.8	0.0	BLOQ	0.0	0.0	BLOQ	BLOQ	BLOQ
U029	SYL ATR	04.19	108	33.9	BLOQ	BLOQ	BLOQ	0.0	0.0	BLOQ	70.4	0.0	BLOQ	0.0	0.0	BLOQ	BLOQ	0.0
U030	SYL ATR	04.19	108	40.1	BLOQ	BLOQ	BLOQ	0.0	0.0	BLOQ	75.7	0.0	0.0	0.0	0.0	BLOQ	0.0	0.0
U031	SYL ATR	04.19	108	18.3	BLOQ	BLOQ	BLOQ	0.0	0.0	BLOQ	8.8	0.0	BLOQ	0.0	0.0	0.0	0.0	0.0
U032	SYL ATR	04.19	108	799.8	61.9	BLOQ	BLOQ	135.5	176.2	BLOQ	248.4	0.0	BLOQ	0.0	0.0	BLOQ	BLOQ	BLOQ
U034	SYL ATR	04.19	108	36.2	7.1	BLOQ	19.2	0.0	0.0	BLOQ	29.8	0.0	BLOQ	0.0	0.0	BLOQ	BLOQ	BLOQ
U035	SYL ATR	04.19	108	148.9	27.3	BLOQ	83.2	BLOQ	0.0	BLOQ	33.2	0.0	BLOQ	0.0	0.0	BLOQ	BLOQ	0.0
U036	LUS MEG	05.20	139	28.0	BLOQ	BLOQ	42.1	0.0	0.0	BLOQ	51.8	0.0	62.9	0.0	0.0	46.0	BLOQ	BLOQ
U037	SYL ATR	05.20	139	109.3	BLOQ	BLOQ	BLOQ	0.0	0.0	BLOQ	65.4	0.0	BLOQ	0.0	0.0	0.0	BLOQ	0.0
U038	SYL ATR	05.20	139	241.8	52.9	175.2	182.0	87.7	120.6	BLOQ	133.8	0.0	BLOQ	0.0	0.0	BLOQ	BLOQ	0.0
U040	SYL ATR	05.20	139	311.8	23.5	79.4	83.2	45.2	58.3	BLOQ	71.8	0.0	BLOQ	0.0	0.0	26.8	BLOQ	BLOQ
U041	SYL ATR	05.30	149	57.5	BLOQ	47.1	0.0	0.0	0.0	BLOQ	0.0	0.0	BLOQ	0.0	0.0	BLOQ	BLOQ	0.0
U042	TUR PHI	05.30	149	41.9	BLOQ	BLOQ	BLOQ	0.0	0.0	BLOQ	19.5	0.0	0.0	0.0	0.0	0.0	BLOQ	0.0
U043	TUR MER	05.30	149	58.9	BLOQ	BLOQ	BLOQ	0.0	0.0	BLOQ	35.1	0.0	0.0	0.0	0.0	BLOQ	0.0	0.0
U044	ACR SCH	05.30	149	913.6	BLOQ	BLOQ	BLOQ	447.7	532.5	BLOQ	419.6	0.0	BLOQ	0.0	0.0	BLOQ	BLOQ	BLOQ

U045	SYL ATR	05.30	149	BLOQ	BLOQ	BLOQ	BLOQ	0.0	0.0	BLOQ	32.5	0.0	0.0	0.0	0.0	BLOQ	0.0	0.0
U046	ACR SCI	05.30	149	752.2	189.0	BLOQ	BLOQ	0.0	0.0	BLOQ	207.7	0.0	BLOQ	0.0	0.0	BLOQ	BLOQ	BLOQ
U047	SYL ATR	05.30	149	293.5	114.2	BLOQ	99.6	0.0	0.0	BLOQ	106.7	0.0	BLOQ	0.0	0.0	BLOQ	BLOQ	BLOQ
U048	ACR SCI	05.30	149	108.6	BLOQ	BLOQ	BLOQ	0.0	0.0	BLOQ	138.9	0.0	BLOQ	0.0	0.0	BLOQ	BLOQ	0.0
U049	ACR SCI	05.30	149	426.9	233.9	BLOQ	BLOQ	0.0	0.0	BLOQ	206.3	0.0	BLOQ	91.5	0.0	122.7	BLOQ	0.0
U050	ACR SCI	05.30	149	72.2	BLOQ	BLOQ	BLOQ	BLOQ	0.0	BLOQ	56.7	0.0	BLOQ	0.0	0.0	BLOQ	BLOQ	0.0
U052	ACR SCI	05.30	149	198.0	36.4	120.9	BLOQ	84.9	98.3	BLOQ	94.9	0.0	BLOQ	0.0	0.0	BLOQ	BLOQ	BLOQ
U053	ACR SCI	05.30	149	233.8	48.0	151.3	92.5	84.3	117.3	BLOQ	70.3	0.0	BLOQ	0.0	0.0	BLOQ	BLOQ	BLOQ
U054	TUR PHI	05.30	149	848.1	144.7	471.9	934.5	363.0	421.3	BLOQ	335.5	0.0	BLOQ	0.0	0.0	BLOQ	BLOQ	BLOQ
U055	TUR MER	05.30	149	26.6	4.8	BLOQ	BLOQ	0.0	0.0	BLOQ	10.3	0.0	BLOQ	0.0	0.0	BLOQ	0.0	BLOQ
U056	ACR ARU	06.08	158	231.9	20.4	BLOQ	BLOQ	59.6	73.7	BLOQ	55.2	0.0	0.0	0.0	0.0	BLOQ	BLOQ	BLOQ
U058	LOC LUS	07.07	187	75.0	9.5	34.0	BLOQ	BLOQ	0.0	24.5	48.5	BLOQ	BLOQ	BLOQ	0.0	101.6	BLOQ	BLOQ
U059	ACR ARU	07.07	187	575.5	43.5	BLOQ	BLOQ	84.8	BLOQ	169.7	195.7	0.0	BLOQ	0.0	0.0	77.4	BLOQ	BLOQ
U060	SYL ATR	07.07	187	2079.1	68.5	BLOQ	BLOQ	0.0	159.1	BLOQ	216.8	0.0	BLOQ	0.0	0.0	BLOQ	BLOQ	BLOQ
U061	LOC LUS	07.07	187	1011.4	205.4	707.2	BLOQ	399.4	529.4	BLOQ	302.1	0.0	BLOQ	0.0	0.0	BLOQ	BLOQ	0.0
U062	SYL ATR	07.07	187	8829.6	347.1	BLOQ	955.5	0.0	0.0	BLOQ	869.7	0.0	BLOQ	0.0	0.0	BLOQ	BLOQ	BLOQ
U063	SYL ATR	07.07	187	2351.2	77.0	281.6	256.1	205.4	237.6	BLOQ	262.1	0.0	BLOQ	BLOQ	0.0	BLOQ	BLOQ	BLOQ
U064	unknown			12268.8	118.9	BLOQ	BLOQ	0.0	897.2	244.6	789.9	BLOQ	BLOQ	1121.0	BLOQ	433.3	BLOQ	128.7
U065	ACR SCH	07.07	187	188.9	BLOQ	BLOQ	BLOQ	71.1	0.0	BLOQ	85.7	0.0	0.0	0.0	0.0	BLOQ	BLOQ	0.0
U066	ACR SCH	07.07	187	3043.5	29.8	BLOQ	BLOQ	60.6	0.0	BLOQ	150.1	0.0	BLOQ	0.0	0.0	BLOQ	BLOQ	BLOQ
U067	LOC LUS	07.07	187	194.5	BLOQ	BLOQ	BLOQ	0.0	0.0	BLOQ	110.7	0.0	0.0	0.0	0.0	BLOQ	BLOQ	0.0
U068	ACR ARU	07.07	187	BLOQ	BLOQ	BLOQ	BLOQ	34.8	0.0	BLOQ	32.6	0.0	BLOQ	0.0	0.0	BLOQ	BLOQ	0.0
U069	LOC LUS	07.07	187	0.0	BLOQ	BLOQ	BLOQ	0.0	0.0	BLOQ	83.9	0.0	BLOQ	0.0	0.0	BLOQ	0.0	0.0
U070	SYL ATR	07.07	187	40.9	BLOQ	BLOQ	BLOQ	0.0	0.0	BLOQ	13.4	0.0	0.0	0.0	0.0	0.0	BLOQ	0.0
U071	LUS MEG	08.03	215	198.0	BLOQ	BLOQ	BLOQ	218.6	219.6	43.7	558.3	BLOQ	BLOQ	583.4	138.1	294.1	0.0	15.4
U072	SYL ATR	08.03	215	36.8	0.0	BLOQ	BLOQ	59.4	61.2	BLOQ	211.0	BLOQ	BLOQ	185.9	BLOQ	81.3	0.0	BLOQ
U073	ACR PAL	08.03	215	1873.4	36.0	BLOQ	BLOQ	3282.9	3113.0	97.5	3593.7	1759.8	BLOQ	3949.8	1773.8	3074.2	0.0	BLOQ
U074	ACR SCI	08.03	215	104.1	0.0	BLOQ	BLOQ	141.3	140.5	0.0	261.9	BLOQ	BLOQ	267.7	BLOQ	147.2	0.0	BLOQ
U075	SYL ATR	08.03	215	2445.1	BLOQ	BLOQ	BLOQ	1543.2	1356.4	BLOQ	1479.2	926.2	BLOQ	2013.5	2676.3	0.0	0.0	BLOQ
U076	SYL ATR	08.03	215	316.9	BLOQ	BLOQ	BLOQ	373.8	396.7	BLOQ	1190.4	BLOQ	BLOQ	1249.7	BLOQ	464.6	0.0	BLOQ
U077	TUR PHI	08.03	215	81.7	BLOQ	BLOQ	BLOQ	BLOQ	0.0	BLOQ	0.0	0.0	0.0	1197.2	BLOQ	14.3	0.0	0.0
U078	LOC LUS	08.03	215	375.2	BLOQ	BLOQ	BLOQ	408.4	428.8	BLOQ	1123.6	BLOQ	BLOQ	1120.6	227.3	547.0	0.0	BLOQ
U079	ACR PAL	08.04	216	68.9	0.0	0.0	BLOQ	39.3	41.5	0.0	84.2	BLOQ	BLOQ	127.7	BLOQ	48.1	0.0	0.0
U080	ERI RUB	08.04	216	1797.0	BLOQ	56.9	BLOQ	4083.9	4035.4	158.2	15783.2	2836.1	BLOQ	25581.9	1410.7	3703.0	0.0	BLOQ
U081	ACR PAL	08.04	216	369.8	BLOQ	BLOQ	BLOQ	862.1	870.2	BLOQ	3581.8	587.3	BLOQ	5564.2	BLOQ	688.1	0.0	0.0
U082	SYL ATR	08.04	216	198.3	BLOQ	BLOQ	BLOQ	550.6	564.1	BLOQ	3066.3	466.0	BLOQ	5309.9	BLOQ	618.2	0.0	0.0
U083	ACR ARU	08.04	216	997.5	BLOQ	BLOQ	BLOQ	2497.3	2459.9	115.0	7594.6	1832.6	BLOQ	13272.0	640.6	2184.0	0.0	0.0
U084	LOC LUS	08.04	216	303.4	0.0	BLOQ	BLOQ	303.0	307.3	0.0	1205.3	BLOQ	BLOQ	2269.8	68.7	296.1	0.0	0.0
U085	LAN COL	08.04	216	1038.0	BLOQ	BLOQ	BLOQ	1369.4	1296.3	0.0	3065.9	BLOQ	BLOQ	5352.1	524.6	1233.8	0.0	0.0
U086	ERI RUB	08.05	217	129.0	0.0	BLOQ	BLOQ	242.0	242.4	BLOQ	924.3	202.3	BLOQ	1967.8	76.5	236.2	0.0	0.0
U087	LUS MEG	08.04	216	103.3	0.0	BLOQ	BLOQ	75.6	80.3	0.0	302.7	BLOQ	BLOQ	643.1	34.1	95.1	0.0	0.0
U088	LOC LUS	08.05	217	694.2	BLOQ	BLOQ	BLOQ	1038.4	981.5	106.1	2668.0	807.8	BLOQ	4852.8	433.2	1022.9	0.0	0.0
U090	ACR SCH	08.05	217	43.1	0.0	BLOQ	BLOQ	26.0	28.4	147.9	50.0	BLOQ	BLOQ	94.2	BLOQ	34.0	0.0	0.0
U091	TUR MER	08.05	217	1215.5	180.4	688.1	BLOQ	499.4	567.1	428.5	382.8	0.0	BLOQ	139801.8	0.0	2634.5	BLOQ	BLOQ
U092	LOC LUS	08.05	217	469.9	0.0	BLOQ	BLOQ	357.5	396.7	BLOQ	887.9	BLOQ	BLOQ	2398.1	BLOQ	367.4	0.0	0.0
U093	ACR SCI	08.05	217	633.9	BLOQ	BLOQ	BLOQ	1059.9	1122.9	BLOQ	2867.0	687.7	BLOQ	6698.6	BLOQ	967.6	0.0	0.0
U094	ACR SCI	08.05	217	289.8	0.0	BLOQ	BLOQ	462.2	546.0	BLOQ	2122.5	BLOQ	BLOQ	5521.0	BLOQ	471.2	0.0	0.0
U095	ERI RUB	08.05	217	267.9	BLOQ	BLOQ	0.0	281.3	326.9	BLOQ	444.6	263.8	BLOQ	116.5	204.4	259.3	0.0	0.0
U096	SYL ATR	08.05	217	35.0	BLOQ	BLOQ	BLOQ	0.0	0.0	BLOQ	0.0	0.0	BLOQ	28.7	0.0	BLOQ	0.0	0.0
U097	SYL ATR	08.05	217	41.0	BLOQ	BLOQ	BLOQ	0.0	0.0	BLOQ	0.0	0.0	0.0	34.4	0.0	BLOQ	0.0	0.0
U098	ERI RUB	08.05	217	50.9	BLOQ	BLOQ	BLOQ	19.9	23.9	BLOQ	0.0	0.0	BLOQ	BLOQ	0.0	BLOQ	BLOQ	0.0
U099	ACR ARU	08.05	217	132.6	BLOQ	BLOQ	BLOQ	38.6	51.4	BLOQ	0.0	0.0	BLOQ	72.2	0.0	BLOQ	BLOQ	0.0
U100	SYL ATR	08.05	217	96.9	33.3	BLOQ	BLOQ	24.8	32.8	BLOQ	0.0	0.0	BLOQ	52.6	0.0	BLOQ	BLOQ	0.0
U101	SYL ATR	08.05	217	14.4	BLOQ	BLOQ	BLOQ	0.0	0.0	BLOQ	0.0	0.0	BLOQ	BLOQ	0.0	BLOQ	BLOQ	0.0
U102	ACR PAL	08.06	218	27.3	BLOQ	BLOQ	BLOQ	0.0	0.0	BLOQ	0.0	0.0	BLOQ	BLOQ	0.0	25.2	BLOQ	0.0
U103	ACR SCI	08.06	218	21.0	BLOQ	BLOQ	BLOQ	0.0	0.0	BLOQ	0.0	0.0	BLOQ	0.0	0.0	BLOQ	BLOQ	0.0
U104	LUS MEG	08.06	218	22.5	BLOQ	BLOQ	BLOQ	0.0	0.0	BLOQ	0.0	0.0	BLOQ	0.0	0.0	BLOQ	BLOQ	0.0
U105	TUR MER	08.06	218	0.0	BLOQ	BLOQ	BLOQ	0.0	0.0	BLOQ	0.0	0.0	0.0	0.0	0.0	BLOQ	0.0	0.0
U106	COT COT	08.06	218	7.4	BLOQ	BLOQ	BLOQ	0.0	0.0	BLOQ	29.7	0.0	BLOQ	14.3	0.0	BLOQ	BLOQ	0.0
U108	LUS MEG	08.06	218	49.4	BLOQ	BLOQ	BLOQ	0.0	0.0	BLOQ	0.0	0.0	BLOQ	0.0	0.0	BLOQ	BLOQ	0.0

U109	LOC LUS	08.06	218	68.2	BLOQ	BLOQ	BLOQ	BLOQ	0.0	BLOQ	0.0	0.0	BLOQ	0.0	0.0	BLOQ	BLOQ	0.0
U110	TUR MER	08.06	218	12.6	BLOQ	BLOQ	BLOQ	0.0	0.0	BLOQ	0.0	0.0	0.0	0.0	0.0	BLOQ	0.0	0.0
U111	SYL ATR	08.06	218	15.2	BLOQ	BLOQ	BLOQ	0.0	0.0	BLOQ	0.0	0.0	BLOQ	0.0	0.0	BLOQ	BLOQ	0.0
U112	LUS MEG	08.06	218	288.9	BLOQ	BLOQ	BLOQ	BLOQ	136.4	BLOQ	0.0	0.0	BLOQ	BLOQ	0.0	BLOQ	BLOQ	0.0
U113	ACR SCI	08.07	219	25.6	BLOQ	BLOQ	BLOQ	0.0	0.0	BLOQ	53.1	0.0	BLOQ	0.0	0.0	BLOQ	BLOQ	0.0
U114	ERI RUB	08.07	219	147.3	BLOQ	BLOQ	BLOQ	62.4	80.5	BLOQ	0.0	0.0	BLOQ	0.0	0.0	BLOQ	BLOQ	0.0
U116	ACR SCH	08.07	219	7.7	BLOQ	BLOQ	BLOQ	0.0	0.0	BLOQ	0.0	0.0	0.0	0.0	0.0	BLOQ	0.0	0.0
U117	SYL ATR	08.07	219	18.8	7.7	BLOQ	BLOQ	9.3	10.1	16.0	0.0	0.0	BLOQ	0.0	0.0	BLOQ	BLOQ	0.0
U118	ERI RUB	08.07	219	16.0	BLOQ	BLOQ	BLOQ	0.0	0.0	BLOQ	0.0	0.0	0.0	0.0	0.0	BLOQ	0.0	0.0
U119	ERI RUB	08.07	219	188.2	BLOQ	BLOQ	BLOQ	BLOQ	110.9	BLOQ	0.0	0.0	BLOQ	0.0	0.0	BLOQ	BLOQ	0.0
U120	LOC LUS	08.07	219	107.3	BLOQ	BLOQ	BLOQ	56.7	74.6	BLOQ	1755.9	0.0	BLOQ	BLOQ	0.0	BLOQ	BLOQ	0.0
U121	LUS MEG	08.08	220	341.7	42.6	BLOQ	BLOQ	130.4	157.3	BLOQ	288.3	0.0	BLOQ	0.0	0.0	108.5	BLOQ	BLOQ
U122	ERI RUB	08.08	220	62.0	BLOQ	BLOQ	BLOQ	0.0	0.0	0.0	1405.1	0.0	74.9	0.0	0.0	BLOQ	0.0	0.0
U123	SYL ATR	08.08	220	130.7	BLOQ	BLOQ	BLOQ	BLOQ	0.0	BLOQ	790.2	0.0	BLOQ	0.0	0.0	BLOQ	BLOQ	0.0
U124	SYL ATR	08.08	220	0.0	BLOQ	BLOQ	BLOQ	0.0	0.0	BLOQ	373.9	0.0	BLOQ	0.0	0.0	0.0	0.0	0.0
U125	LUS MEG	08.08	220	102.5	BLOQ	BLOQ	BLOQ	0.0	0.0	0.0	1645.9	0.0	133.3	0.0	0.0	BLOQ	0.0	0.0
U126	ERI RUB	08.08	220	34.1	BLOQ	BLOQ	BLOQ	14.2	15.6	BLOQ	26.0	0.0	BLOQ	0.0	0.0	BLOQ	BLOQ	BLOQ
U127	ERI RUB	08.08	220	0.0	BLOQ	BLOQ	BLOQ	0.0	0.0	BLOQ	720.5	0.0	64.1	0.0	0.0	BLOQ	0.0	0.0
U128	LOC LUS	08.08	220	37.6	BLOQ	BLOQ	BLOQ	14.3	16.8	BLOQ	30.8	0.0	BLOQ	0.0	0.0	BLOQ	BLOQ	BLOQ
U129	LUS MEG	08.08	220	18.6	BLOQ	BLOQ	BLOQ	0.0	0.0	BLOQ	93.9	0.0	BLOQ	0.0	0.0	BLOQ	0.0	0.0
U130	PAR MAJ	08.08	220	40.4	BLOQ	BLOQ	BLOQ	BLOQ	0.0	BLOQ	0.0	0.0	BLOQ	0.0	0.0	14.7	BLOQ	0.0
U131	ERI RUB	08.08	220	219.4	31.6	BLOQ	BLOQ	58.1	0.0	0.0	701.5	0.0	224.0	0.0	0.0	BLOQ	BLOQ	0.0
U132	SYL COM	08.08	220	118.7	BLOQ	BLOQ	BLOQ	0.0	0.0	BLOQ	310.1	0.0	BLOQ	0.0	0.0	BLOQ	BLOQ	0.0
U134	ACR SCH	08.09	221	84.1	BLOQ	BLOQ	BLOQ	0.0	0.0	BLOQ	114.6	0.0	BLOQ	0.0	0.0	BLOQ	BLOQ	0.0
U135	LOC LUS	08.09	221	348.7	69.7	BLOQ	BLOQ	168.7	203.1	BLOQ	452.4	0.0	BLOQ	0.0	0.0	BLOQ	BLOQ	BLOQ
U136	LUS MEG	08.10	222	0.0	BLOQ	BLOQ	BLOQ	0.0	0.0	BLOQ	127.9	0.0	BLOQ	0.0	0.0	BLOQ	0.0	0.0
U137	LUS LUS	08.10	222	101.4	0.0	BLOQ	BLOQ	0.0	0.0	0.0	239.5	0.0	BLOQ	0.0	0.0	BLOQ	0.0	0.0
U138	ERI RUB	08.17	228	0.0	BLOQ	BLOQ	BLOQ	0.0	0.0	0.0	78.8	0.0	BLOQ	0.0	0.0	0.0	0.0	0.0
U139	ACR SCI	08.17	228	233.0	36.2	BLOQ	BLOQ	115.6	138.3	BLOQ	358.3	0.0	BLOQ	0.0	0.0	BLOQ	BLOQ	0.0
U140	ACR SCI	08.17	228	32.0	BLOQ	BLOQ	BLOQ	0.0	19.5	0.0	115.6	0.0	BLOQ	0.0	0.0	BLOQ	BLOQ	0.0
U141	ACR SCH	08.17	228	141.3	28.6	BLOQ	BLOQ	69.0	104.5	0.0	1266.6	0.0	524.0	0.0	0.0	BLOQ	BLOQ	0.0
U142	ACR SCI	08.18	229	123.4	BLOQ	BLOQ	BLOQ	60.7	76.8	0.0	507.4	0.0	BLOQ	0.0	0.0	BLOQ	BLOQ	BLOQ
U143	ACR SCI	08.18	229	223.6	53.0	BLOQ	BLOQ	106.9	139.0	BLOQ	395.7	0.0	BLOQ	0.0	0.0	BLOQ	BLOQ	BLOQ
U144	ACR SCI	08.19	230	51.2	BLOQ	BLOQ	BLOQ	0.0	0.0	0.0	140.2	0.0	BLOQ	0.0	0.0	BLOQ	BLOQ	BLOQ
U145	LOC LUS	08.20	231	134.2	BLOQ	BLOQ	BLOQ	0.0	0.0	BLOQ	113.0	0.0	BLOQ	0.0	0.0	BLOQ	0.0	0.0
U146	LUS MEG	08.21	232	120.3	19.5	BLOQ	BLOQ	52.3	61.4	BLOQ	114.1	0.0	BLOQ	0.0	0.0	BLOQ	BLOQ	BLOQ
U147	LOC LUS	08.22	233	258.3	BLOQ	BLOQ	BLOQ	BLOQ	0.0	BLOQ	0.0	0.0	BLOQ	BLOQ	0.0	BLOQ	BLOQ	0.0
U148	LUS SVE	08.22	233	49.8	9.7	BLOQ	BLOQ	BLOQ	29.5	BLOQ	168.0	0.0	62.6	0.0	0.0	BLOQ	BLOQ	BLOQ
U149	ERI RUB	08.22	233	120.1	BLOQ	BLOQ	BLOQ	47.0	75.7	0.0	478.3	0.0	123.1	0.0	0.0	BLOQ	BLOQ	BLOQ
U150	ACR SCH	08.23	234	149.6	BLOQ	BLOQ	BLOQ	74.0	89.3	BLOQ	1032.3	0.0	BLOQ	0.0	0.0	BLOQ	BLOQ	0.0
U151	SYL ATR	08.23	234	94.7	BLOQ	BLOQ	BLOQ	BLOQ	0.0	BLOQ	764.5	0.0	BLOQ	0.0	0.0	BLOQ	BLOQ	0.0
Ux01	SYL ATR	09.04	246	0.0	BLOQ	BLOQ	BLOQ	0.0	0.0	0.0	711.3	0.0	BLOQ	0.0	0.0	BLOQ	BLOQ	0.0
Ux02	SYL COM	09.04	246	333.1	BLOQ	BLOQ	BLOQ	93.6	126.5	BLOQ	498.2	0.0	BLOQ	0.0	0.0	BLOQ	BLOQ	0.0
Ux03	SYL COM	09.04	246	61.4	BLOQ	BLOQ	BLOQ	0.0	0.0	BLOQ	605.3	0.0	BLOQ	0.0	0.0	BLOQ	0.0	0.0
Ux04	SYL COM	09.04	246	59.9	BLOQ	BLOQ	BLOQ	0.0	0.0	BLOQ	348.3	0.0	BLOQ	0.0	0.0	BLOQ	BLOQ	0.0
Ux05	ERI RUB	09.04	246	193.9	34.9	BLOQ	BLOQ	82.5	99.4	BLOQ	327.3	0.0	BLOQ	0.0	0.0	BLOQ	BLOQ	0.0
Ux06	TUR MER	09.04	246	19.8	BLOQ	BLOQ	BLOQ	0.0	0.0	BLOQ	185.8	0.0	BLOQ	0.0	0.0	BLOQ	0.0	0.0
Ux07	PAR MAJ	09.04	246	55.3	BLOQ	BLOQ	BLOQ	27.9	0.0	0.0	273.8	0.0	BLOQ	0.0	0.0	BLOQ	BLOQ	0.0
Ux08	SYL ATR	09.05	247	BLOQ	0.0	BLOQ		0.0	0.0	0.0	349.9	0.0	BLOQ	0.0	0.0	BLOQ	0.0	0.0
Ux09	ERI RUB	08.23	234	166.3	BLOQ	0.0	BLOQ	0.0	0.0	0.0	273.9	0.0	BLOQ	0.0	0.0	BLOQ	0.0	0.0
Ux10	ERI RUB	09.05	247	36.5	BLOQ	BLOQ	BLOQ	13.7	23.0	BLOQ	58.3	0.0	BLOQ	0.0	0.0	BLOQ	BLOQ	0.0
Ux11	ERI RUB	09.05	247	75.9	BLOQ	BLOQ	BLOQ	0.0	0.0	BLOQ	43.2	0.0	BLOQ	0.0	0.0	BLOQ	0.0	0.0
Ux12	ERI RUB	09.05	247	402.4	41.1	137.8	132.9	0.0	0.0	BLOQ	114.3	0.0	BLOQ	0.0	0.0	BLOQ	BLOQ	BLOQ
Ux13	TUR PHI	09.05	247	11.6	BLOQ	BLOQ	BLOQ	0.0	0.0	BLOQ	13.3	0.0	BLOQ	0.0	0.0	BLOQ	BLOQ	0.0
Ux14	SYL COM	09.06	248	76.2	BLOQ	BLOQ	BLOQ	33.4	40.1	BLOQ	122.7	0.0	BLOQ	0.0	0.0	BLOQ	BLOQ	0.0
Ux15	ERI RUB	09.07	249	144.3	BLOQ	BLOQ	BLOQ	0.0	0.0	BLOQ	126.6	0.0	0.0	0.0	0.0	BLOQ	0.0	0.0
Ux16	ERI RUB	09.07	249	67.9	BLOQ	BLOQ	BLOQ	0.0	0.0	BLOQ	173.3	0.0	BLOQ	0.0	0.0	BLOQ	0.0	0.0
Ux17	ERI RUB	09.07	249	94.5	BLOQ	BLOQ	BLOQ	0.0	0.0	BLOQ	140.9	0.0	BLOQ	0.0	0.0	BLOQ	BLOQ	0.0
Ux31	PRU MOD	10.18	290	146.1	BLOQ	BLOQ	BLOQ	0.0	0.0	BLOQ	85.7	0.0	BLOQ	0.0	0.0	BLOQ	0.0	0.0
Ux32	ERI RUB	10.18	290	59.5	0.0	0.0	BLOQ	0.0	0.0	BLOQ	106.9	0.0	BLOQ	0.0	0.0	0.0	0.0	0.0

Ux33	PAR MAJ	10.18	290	134.7	28.0	BLOQ	BLOQ	59.2	75.8	BLOQ	91.3	0.0	BLOQ	0.0	0.0	BLOQ	BLOQ	0.0
Ux34	TUR MER	10.18	290	147.6	26.8	BLOQ	BLOQ	64.0	82.9	BLOQ	77.4	0.0	BLOQ	0.0	0.0	BLOQ	BLOQ	BLOQ
Ux36	ERI RUB	10.18	290	68.5	BLOQ	BLOQ	BLOQ	0.0	0.0	BLOQ	113.8	0.0	BLOQ	0.0	0.0	BLOQ	0.0	BLOQ
Ux37	ERI RUB	10.18	290	76.1	BLOQ	BLOQ	BLOQ	37.3	49.6	BLOQ	50.1	0.0	BLOQ	0.0	0.0	BLOQ	BLOQ	BLOQ
Ux38	PRU MOD	10.18	290	33.7	3.2	9.1	11.3	0.0	0.0	3.3	9.6	0.0	BLOQ	0.0	0.0	1.8	BLOQ	BLOQ
Ux39	ERI RUB	10.18	290	0.0	0.0	BLOQ	BLOQ	0.0	0.0	BLOQ	17.4	0.0	BLOQ	0.0	0.0	BLOQ	0.0	0.0
Ux40	PRU MOD	10.18	290	0.0	BLOQ	BLOQ	BLOQ	0.0	0.0	BLOQ	8.6	0.0	BLOQ	0.0	0.0	BLOQ	0.0	0.0
Ux41	PRU MOD	10.18	290	4.0	BLOQ	BLOQ	BLOQ	0.0	0.0	BLOQ	8.7	0.0	BLOQ	0.0	0.0	2.0	BLOQ	BLOQ
Ux42	ERI RUB	10.18	290	BLOQ	0.0	BLOQ	BLOQ	0.0	0.0	BLOQ	13.6	0.0	BLOQ	0.0	0.0	BLOQ	0.0	0.0

Table S6. Data according to bird species: dates of sampling and corresponding blood concentrations of ecdysteroids **1-15** (ng/mL). For abbreviations of bird species see Fig 1.

No.	BIRD SP	DATE	MONTH	DAY/YEAR	20E (1)	20E 2-acetate (2)	20E 3-acetate (3)	20E 20,22-acetonide (4)	2-Deoxy-20E (5)	Ajugasterone C (6)	Calonysterone (7)	Dacryhainansterone (8)	Ecdysone (9)	9,11-Didehydro-poststerone (10)	2-Deoxy-poststerone (11)	Polypodine B (12)	Poststerone (13)	Rubrosterone (14)	Shidasterone (15)
S02	ERI RUB	03.21	MARC	79	431.0	404.8	1350.9	89.9			315.4	57.3	0.1				153.5	31.8	59.9
S06	ERI RUB	03.21	MARC	79	173.4	41.2					211.5	82.0		35.4			246.9		174.1
S07	ERI RUB	03.21	MARC	79	210.8	90.9	286.4	202.1	733.7	452.0	686.3	171.7		135.5			960.7		1214.5
S08	ERI RUB	03.21	MARC	79	74.6				176.5	114.5	158.2	59.9		57.0			243.4		338.7
S11	ERI RUB	03.21	MARC	79	94.4			52.9	53.9	55.2	99.0	49.8							57.5
S12	ERI RUB	03.21	MARC	79	197.9				82.7	90.1	137.3	98.4							215.7
S15	ERI RUB	03.21	MARC	79	167.0	36.3		147.5	73.2	120.2	143.4	140.0							249.5
S16	ERI RUB	03.21	MARC	79	26.4			25.4	15.5	24.3	23.0	44.0							53.2
S17	ERI RUB	03.21	MARC	79	70.7	15.4		42.4	43.7	59.0	58.1	55.6							73.8
S22	ERI RUB	03.22	MARC	80	44.6		49.9		25.5	42.2		19.7					23.8		
S24	ERI RUB	03.22	MARC	80	162.0	48.1				239.1	96.6	98.1							
S25	ERI RUB	03.22	MARC	80						7.1									
S33	ERI RUB	03.22	MARC	80	41.4	7.6				31.7									
S38	ERI RUB	03.29	MARC	87	43.0				11.1										
S39	ERI RUB	03.29	MARC	87	58.4	12.1				102.1	30.0								
S40	ERI RUB	03.29	MARC	87		11.4			21.5	41.9	30.8								
S43	ERI RUB	03.29	MARC	87															
S47	ERI RUB	03.29	MARC	87	281.1	50.1	157.7	132.3	106.8	126.4		149.1							
S48	ERI RUB	03.29	MARC	87	31.8				12.4	15.3		24.3							
S49	ERI RUB	03.29	MARC	87	33.8							24.9							
S50	ERI RUB	03.29	MARC	87															
S51	ERI RUB	03.29	MARC	87	34.0														
S52	ERI RUB	03.29	MARC	87															
S53	ERI RUB	03.29	MARC	87	643.6	59.8	199.0		110.4	130.0		155.1							
S55	ERI RUB	03.29	MARC	87	5.7														
S56	ERI RUB	03.29	MARC	87	18.4														
S57	ERI RUB	03.29	MARC	87															
S58	ERI RUB	03.29	MARC	87	67.4		31.4		36.9	41.2		30.7							
S59	ERI RUB	03.29	MARC	87	818.9				174.2	154.0		141.6		141.4			401.1		
S60	ERI RUB	03.29	MARC	87	16.4					7.8	28.4	110.4							
S63	ERI RUB	03.29	MARC	87	22.4				7.6	8.8	11.6	103.9		14.7					
S64	ERI RUB	03.29	MARC	87	39.2						38.6	517.1							
S65	ERI RUB	03.29	MARC	87	235.4				116.9	186.0		1211.8		206.2					
S67	ERI RUB	03.29	MARC	87	58.1					28.8									
S69	ERI RUB	03.29	MARC	87	736.3	116.5	369.2		301.1	356.7		2603.1		431.6					
S70	ERI RUB	03.29	MARC	87								41.3							
S72	ERI RUB	03.29	MARC	87	797.7				242.3	292.2		437.8							
S73	ERI RUB	03.29	MARC	87	318.4				92.4		148.3	623.2							
S74	ERI RUB	03.29	MARC	87	162.6			48.9	42.2	45.5	76.4	240.6							
S75	ERI RUB	04.07	APRIL	96	82.7				36.3			321.0		77.5					
S77	ERI RUB	04.07	APRIL	96								433.7							
S78	ERI RUB	04.08	APRIL	97	93.4				44.2	47.7									
U001	ERI RUB	04.18	APRIL	107	37.0						15.8	47.7					10.0		
U002	ERI RUB	04.18	APRIL	107	2.6				2.1			3.7					5.8		
U012	ERI RUB	04.19	APRIL	108								5.9							
U023	ERI RUB	04.19	APRIL	108								184.5							
U080	ERI RUB	08.04	AUGUST	216	1797.0		56.9		4083.9	4035.4	158.2	15783.2	2836.1		ALOQ	1410.7	3703.0		
U086	ERI RUB	08.05	AUGUST	217	129.0				242.0	242.4		924.3	202.3		1967.8	76.5	236.2		
U095	ERI RUB	08.05	AUGUST	217	267.9				281.3	326.9		444.6	263.8		116.5	204.4	259.3		
U098	ERI RUB	08.05	AUGUST	217	50.9				19.9	23.9									
U114	ERI RUB	08.07	AUGUST	219	147.3				62.4	80.5									
U118	ERI RUB	08.07	AUGUST	219	16.0														
U119	ERI RUB	08.07	AUGUST	219	188.2					110.9									
U122	ERI RUB	08.08	AUGUST	220	62.0							1405.1		74.9					
U126	ERI RUB	08.08	AUGUST	220	34.1				14.2	15.6		26.0							
U127	ERI RUB	08.08	AUGUST	220								720.5		64.1					
U131	ERI RUB	08.08	AUGUST	220	219.4	31.6			58.1			701.5		224.0					
U138	ERI RUB	08.17	AUGUST	228								78.8							
U149	ERI RUB	08.22	AUGUST	233	120.1				47.0	75.7		478.3		123.1					
Ux05	ERI RUB	09.04	SEPTEMBE	246	193.9	34.9			82.5	99.4		327.3							
Ux09	ERI RUB	08.23	AUGUST	234	166.3							273.9							
Ux10	ERI RUB	09.05	SEPTEMBE	247	36.5				13.7	23.0		58.3							
Ux11	ERI RUB	09.05	SEPTEMBE	247	75.9							43.2							
Ux12	ERI RUB	09.05	SEPTEMBE	247	402.4	41.1	137.8	132.9				114.3							
Ux15	ERI RUB	09.07	SEPTEMBE	249	144.3							126.6							
Ux16	ERI RUB	09.07	SEPTEMBE	249	67.9							173.3							
Ux17	ERI RUB	09.07	SEPTEMBE	249	94.5							140.9							
Ux32	ERI RUB	10.18	OCTOBER	290	59.5							106.9							
Ux36	ERI RUB	10.18	OCTOBER	290	68.5							113.8							
Ux37	ERI RUB	10.18	OCTOBER	290	76.1				37.3	49.6		50.1							
Ux39	ERI RUB	10.18	OCTOBER	290								17.4							
Ux42	ERI RUB	10.18	OCTOBER	290								13.6							

No.	BIRD SP	DATE	MONTH	DAY/YEAR	20E (1)	20E 2-acetate	20E 3-acetate	2-Deoxy-20E (5)	Ajugasterone C (6)	Dacryhainansterone (8)	2-Deoxy-poststerone (11)	Poststerone (13)
U006	ACR SCI	04.18	APRIL	107	670.5					1642.3		916.9
U026	ACR SCI	04.19	APRIL	108	46.8					117.2		
U028	ACR SCI	04.19	APRIL	108	392.6	66.6	227.8	187.2	204.36	403.8		
U046	ACR SCI	05.30	MAY	149	752.2	189.0				207.7		
U048	ACR SCI	05.30	MAY	149	108.6					138.9		
U049	ACR SCI	05.30	MAY	149	426.9	233.9				206.3	91.5	122.7
U050	ACR SCI	05.30	MAY	149	72.2					56.7		
U052	ACR SCI	05.30	MAY	149	198.0	36.4	120.9	84.9	98.3	94.7		
U053	ACR SCI	05.30	MAY	149	233.8	48.0	151.3	84.3	117.3	70.3		
U074	ACR SCI	08.03	AUGUST	215	104.1			141.3	140.5	261.9	267.7	147.2
U093	ACR SCI	08.05	AUGUST	217	633.9			1059.9	1122.9	2867.0	ALOQ	967.6
U094	ACR SCI	08.05	AUGUST	217	289.8			462.2	546.0	2122.5	5521.0	471.2
U103	ACR SCI	08.06	AUGUST	218	21.0							
U113	ACR SCI	08.07	AUGUST	219	25.6					53.1		
U139	ACR SCI	08.17	AUGUST	228	233.0	36.2		115.6	138.3	358.3		
U140	ACR SCI	08.17	AUGUST	228	32.0				19.5	115.6		
U142	ACR SCI	08.18	AUGUST	229	123.4			60.7	76.8	507.4		
U143	ACR SCI	08.18	AUGUST	229	223.6	53.0		106.9	139.0	395.7		
U144	ACR SCI	08.19	AUGUST	230	51.2					140.2		

No.	BIRD SP	DATE	MONTH	DAY/YEAR	20E (1)	20E 2-acetate (2)	20E 3-acetate (3)	2-Deoxy-20E (5)	Ajugasterone C (6)	Calonysterone (7)	Dacryhainansterone (8)	2-Deoxy-poststerone (11)	Polypodine B (12)	Poststerone (13)
U058	LOC LUS	07.07	JULY	187	75.0	9.5	34.0			24.5	48.5			101.6
U061	LOC LUS	07.07	JULY	187	1011.4	205.4	707.2	399.4	529.4		302.1			
U067	LOC LUS	07.07	JULY	187	194.5						110.7			
U069	LOC LUS	07.07	JULY	187							83.9			
U078	LOC LUS	08.03	AUGUST	215	375.2			408.4	428.8		1123.6	1120.6	227.3	547.0
U084	LOC LUS	08.04	AUGUST	216	303.4			303.0	307.3		1205.3	2269.8	68.7	296.1
U088	LOC LUS	08.05	AUGUST	217	694.2			1038.4	981.5	106.1	2668.0	ALOQ	433.2	1022.9
U092	LOC LUS	08.05	AUGUST	217	469.9			357.5	396.7		887.9	2398.1		367.4
U109	LOC LUS	08.06	AUGUST	218	68.2									
U120	LOC LUS	08.07	AUGUST	219	107.3			56.7	74.6		1755.9			
U128	LOC LUS	08.08	AUGUST	220	37.6			14.3	16.8		30.8			
U135	LOC LUS	08.09	AUGUST	221	348.7	69.7		168.7	203.1		452.4			
U145	LOC LUS	08.20	AUGUST	231	134.2						113.0			
U147	LOC LUS	08.22	AUGUST	233	258.3									

No.	BIRD SP	DATE	MONTH	DAY/YEAR	20E (1)	20E 2-acetate (2)	20E 3-acetate (3)	20E 20,22-acetonide (4)	2-Deoxy-20E (5)	Ajugasterone C (6)	Calonysterone (7)	Dacryhainansterone (8)	Poststerone (13)	Shidasterone (15)
S03	PRU MOD	03.21	MARCH	79	147.6	31.8		102.9			141.7	43.3	35.5	47.8
S19	PRU MOD	03.22	MARCH	80					5.7			7.6		14.9
S26	PRU MOD	03.22	MARCH	80										
S27	PRU MOD	03.22	MARCH	80	123.6	20.2		26.8		176.7	51.9	35.1	30.9	24.6
S28	PRU MOD	03.22	MARCH	80	22.8				11.6	21.5	8.1	9.4		
S29	PRU MOD	03.22	MARCH	80						3.6				
S34	PRU MOD	03.22	MARCH	80	89.6					39.1		34.0		38.6
S37	PRU MOD	03.22	MARCH	80	343.2				106.6			168.6		
S42	PRU MOD	03.29	MARCH	87	593.1	45.8	162.9	121.9	116.7	143.3		148.0		
S44	PRU MOD	03.29	MARCH	87	25.3					16.5		18.4		
S46	PRU MOD	03.29	MARCH	87	197.9							126.3		
S62	PRU MOD	03.29	MARCH	87	322.2				119.6			171.6	96.7	
S66	PRU MOD	03.29	MARCH	87	431.6	64.0		185.4	201.5	227.2		1302.3		
S71	PRU MOD	03.29	MARCH	87	481.7				89.8	104.0	152.4	199.2	108.3	
S76	PRU MOD	04.07	APRIL	96	490.1				164.2	171.9		208.1	148.4	
Ux31	PRU MOD	10.18	OCTOBER	290	146.1							85.7		
Ux38	PRU MOD	10.18	OCTOBER	290	33.7	3.2	9.1	11.3			3.3	9.6	1.8	
Ux40	PRU MOD	10.18	OCTOBER	290								8.6		
Ux41	PRU MOD	10.18	OCTOBER	290	4.0							8.7	2.0	

No.	BIRD SP	DATE	MONTH	DAY/YEAR	20E (1)	20E 2-acetate (2)	20E 3-acetate (3)	20E 20,22-acetonide (4)	2-Deoxy-20E (5)	Ajugasterone C (6)	Calonysterone (7)	Dacryhainansterone (8)	Ecdysone (9)	9,11-Didehydro-poststerone (10)	2-Deoxy-poststerone (11)	Poststerone (13)
U004	SYL ATR	04.18	APRIL	107	6.9				4.4			126.7		9.7		
U005	SYL ATR	04.18	APRIL	107	196.4				78.5			860.4				
U007	SYL ATR	04.18	APRIL	107	963.7	55.1	190.2			128.6		668.7				91.2
U008	SYL ATR	04.18	APRIL	107	14.2				9.0	9.4		247.7		26.4		11.1
U010	SYL ATR	04.18	APRIL	107	15.8							275.5		20.0		
U011	SYL ATR	04.19	APRIL	108	26.2							32.8				
U013	SYL ATR	04.19	APRIL	108								11.3				
U015	SYL ATR	04.19	APRIL	108	9.1							12.5				
U019	SYL ATR	04.19	APRIL	108							39.3	1273.7		67.5		
U020	SYL ATR	04.19	APRIL	108	56.0							500.3				
U022	SYL ATR	04.19	APRIL	108	44.9	10.5	36.5		25.2	47.3		25.0				
U024	SYL ATR	04.19	APRIL	108	150.2						69.2	58.0				38.5
U025	SYL ATR	04.19	APRIL	108	190.9	22.2	83.6		63.1	74.0		119.4				
U027	SYL ATR	04.19	APRIL	108	60.6	10.0						61.1				
U029	SYL ATR	04.19	APRIL	108	33.9							70.4				
U030	SYL ATR	04.19	APRIL	108	40.1							75.7				
U031	SYL ATR	04.19	APRIL	108	18.3							8.8				
U032	SYL ATR	04.19	APRIL	108	799.8	61.9			135.5	176.2		248.4				
U034	SYL ATR	04.19	APRIL	108	36.2	7.1		19.2				29.8				
U035	SYL ATR	04.19	APRIL	108	148.9	27.3		83.2				33.2				
U037	SYL ATR	05.20	MAJUS	139	109.3							65.4				
U038	SYL ATR	05.20	MAJUS	139	241.8	52.9	175.2	182.0	87.7	120.6		133.8				
U040	SYL ATR	05.20	MAJUS	139	311.8	23.5	79.4	83.2	45.2	58.3		71.8				26.8
U041	SYL ATR	05.30	MAY	149	57.5		47.1									
U045	SYL ATR	05.30	MAY	149								32.5				
U047	SYL ATR	05.30	MAY	149	293.5	114.2		99.6				106.7				
U060	SYL ATR	07.07	JULY	187	2079.1	68.5				159.1		216.8				
U062	SYL ATR	07.07	JULY	187	8829.6	347.1		955.5				869.7				
U063	SYL ATR	07.07	JULY	187	2351.2	77.0	281.6	256.1	205.4	237.6		262.1				
U070	SYL ATR	07.07	JULY	187	40.9							13.4				
U072	SYL ATR	08.03	AUGUST	215	36.8				59.4	61.2		211.0			185.9	81.3
U075	SYL ATR	08.03	AUGUST	215	2445.1				1543.2	1356.4		1479.2	926.2		2013.5	
U076	SYL ATR	08.03	AUGUST	215	316.9				373.8	396.7		1190.4			1249.7	464.6
U082	SYL ATR	08.04	AUGUST	216	198.3				550.6	564.1		3066.3	466.0		ALOQ	618.2
U096	SYL ATR	08.05	AUGUST	217	35.0										28.7	
U097	SYL ATR	08.05	AUGUST	217	41.0										34.4	
U100	SYL ATR	08.05	AUGUST	217	96.9	33.3			24.8	32.8					52.6	
U101	SYL ATR	08.05	AUGUST	217	14.4											
U111	SYL ATR	08.06	AUGUST	218	15.2											
U117	SYL ATR	08.07	AUGUST	219	18.8	7.7			9.3	10.1	16.0					
U123	SYL ATR	08.08	AUGUST	220	130.7							790.2				
U124	SYL ATR	08.08	AUGUST	220								373.9				
U151	SYL ATR	08.23	AUGUST	234	94.7							764.5				
Ux01	SYL ATR	09.04	SEPTEMBE	246								711.3				
Ux08	SYL ATR	09.05	SEPTEMBE	247								349.9				

No.	BIRD SP	DATE	MONTH	DAY/YEAR	20E (1)	20E 2-acetate (2)	2-Deoxy-20E (5)	Ajugasterone C (6)	Dacryhainansterone (8)	Poststerone (13)
S21	TUR MER	03.22	MARCH	80	3.6		2.7	4.3		3.4
S45	TUR MER	03.29	MARCH	87	165.9	24.6	67.1	79.4	84.0	65.9
S54	TUR MER	03.29	MARCH	87	16.4				8.1	
S68	TUR MER	03.29	MARCH	87						
S80	TUR MER	04.09	APRIL	98						
U003	TUR MER	04.18	APRIL	107	4.1	4.4				
U017	TUR MER	04.19	APRIL	108					18.2	
U018	TUR MER	04.19	APRIL	108	43.7				1316.1	
U043	TUR MER	05.30	MAY	149	58.9				35.1	
U055	TUR MER	05.30	MAY	149	26.6	4.8			10.3	
U091	TUR MER	08.05	AUGUST	217	1215.5	180.4	499.4	567.1	382.8	2634.5
U105	TUR MER	08.06	AUGUST	218						
U110	TUR MER	08.06	AUGUST	218	12.6					
Ux06	TUR MER	09.04	SEPTEMBEI	246	19.8				185.8	
Ux34	TUR MER	10.18	OCTOBER	290	147.6	26.8	64.0	82.9	77.4	
